# Association of a Safety Program for Improving Antibiotic Use With Antibiotic Use and Hospital-Onset *Clostridioides difficile* Infection Rates Among US Hospitals

**DOI:** 10.1001/jamanetworkopen.2021.0235

**Published:** 2021-02-26

**Authors:** Pranita D. Tamma, Melissa A. Miller, Prashila Dullabh, Roy Ahn, Kathleen Speck, Yue Gao, Erik Scherpf, Sara E. Cosgrove

**Affiliations:** 1 Department of Pediatrics, Johns Hopkins University School of Medicine, Baltimore, Maryland; 2 Center for Quality Improvement and Patient Safety, Agency for Healthcare Research and Quality, Rockville, Maryland; 3 NORC at the University of Chicago, Bethesda, Maryland; 4 NORC at the University of Chicago, Chicago, Illinois; 5 Armstrong Institute for Patient Safety and Quality, Johns Hopkins University School of Medicine, Baltimore, Maryland; 6 Department of Medicine, Johns Hopkins University School of Medicine, Baltimore, Maryland

## Abstract

**Importance:**

Regulatory agencies and professional organizations recommend antibiotic stewardship programs (ASPs) in US hospitals. The optimal approach to establish robust, sustainable ASPs across diverse hospitals is unknown.

**Objective:**

To assess whether the Agency for Healthcare Research and Quality Safety Program for Improving Antibiotic Use is associated with reductions in antibiotic use across US hospitals.

**Design, Setting, and Participants:**

A pragmatic quality improvement program was conducted and evaluated over a 1-year period in US hospitals. A total of 437 hospitals were enrolled. The study was conducted from December 1, 2017, to November 30, 2018. Data analysis was performed from March 1 to October 31, 2019.

**Interventions:**

The Safety Program assisted hospitals with establishing ASPs and worked with frontline clinicians to improve their antibiotic decision-making. All clinical staff (eg, clinicians, pharmacists, and nurses) were encouraged to participate. Seventeen webinars occurred over 12 months, accompanied by additional durable educational content. Topics focused on establishing ASPs, the science of safety, improving teamwork and communication, and best practices for the diagnosis and management of infectious processes.

**Main Outcomes and Measures:**

The primary outcome was overall antibiotic use (days of antibiotic therapy [DOT] per 1000 patient days [PD]) comparing the beginning (January-February 2018) and end (November-December 2018) of the Safety Program. Data analysis occurred using linear mixed models with random hospital unit effects. Antibiotic use from 614 hospitals in the Premier Healthcare Database from the same period was analyzed to evaluate contemporary US antibiotic trends. Quarterly hospital-onset *Clostridioides difficile* laboratory-identified events per 10 000 PD were a secondary outcome.

**Results:**

Of the 437 hospitals enrolled, 402 (92%) remained in the program until its completion, including 28 (7%) academic medical centers, 122 (30%) midlevel teaching hospitals, 167 (42%) community hospitals, and 85 (21%) critical access hospitals. Adherence to key components of ASPs (ie, interventions before and after prescription of antibiotics, availability of local antibiotic guidelines, ASP leads with dedicated salary support, and quarterly reporting of antibiotic use) improved from 8% to 74% over the 1-year period (*P* < .01). Antibiotic use decreased by 30.3 DOT per 1000 PD (95% CI, −52.6 to −8.0 DOT; *P* = .008). Similar changes in antibiotic use were not observed in the Premier Healthcare Database. The incidence rate of hospital-onset *C difficile* laboratory-identified events decreased by 19.5% (95% CI, −33.5% to −2.4%; *P* = .03).

**Conclusions and Relevance:**

The Agency for Healthcare Research and Quality Safety Program appeared to enable diverse hospitals to establish ASPs and teach frontline clinicians to self-steward their antibiotic use. Safety Program content is publicly available.

## Key Points

**Question:**

Is emphasizing improved communication, teamwork, and clinical best practices associated with reductions in antibiotic use across a large number of US hospitals?

**Findings:**

In this quality improvement study of 402 hospitals, implementation of the Agency for Healthcare Research and Quality Safety Program was associated with a reduction in antibiotic use and hospital-onset *Clostridioides difficile* infection rates, including critical access hospitals, rural hospitals, and hospitals without infectious diseases specialists. The greatest reduction in antibiotic use was observed in sites most actively engaged in the Safety Program.

**Meaning:**

The Agency for Healthcare Research and Quality Safety Program resources, now publicly available, may be useful in teaching frontline clinicians to become stewards of their antibiotic use.

## Teaser

This quality improvement study examines the use of the Agency for Healthcare Research and Quality Safety Program for antibiotic stewardship across US hospitals.

## Introduction

Antibiotic stewardship programs (ASPs) are being established across the US in response to the negative consequences associated with antibiotic overuse.^1,2^ Although ASPs have been successful in reducing antibiotic use within institutions,^3^ their ability to train clinicians to self-steward their antibiotic use on an ongoing basis has been less of a focus. Antibiotic stewardship programs are limited in their resources to provide real-time assistance with all antibiotics prescribed in a hospital, leading to lost opportunities to improve antibiotic prescribing. Moreover, teaching clinicians to incorporate stewardship concepts into their clinical practice supports the durability of stewardship principles in daily patient care. In response to drawbacks of the traditional top-down ASP approach, the Agency for Healthcare Research and Quality (AHRQ) established the AHRQ Safety Program for Improving Antibiotic Use (ie, the Safety Program).

In addition to assisting hospitals with establishing sustainable ASPs, an overarching goal of the Safety Program is to provide frontline clinicians with tools to incorporate stewardship principles into routine decision-making by underscoring the importance of communication around antibiotic prescribing and equipping frontline clinicians with best practices in the diagnosis and treatment of common infectious processes. The underpinnings of the Safety Program are largely drawn from the Comprehensive Unit-Based Safety Program method, an approach to make health care safer by emphasizing improved teamwork, clinical best practices, and the science of safety.^4^ We discuss the structure, implementation, and outcomes of the Safety Program in 402 hospitals across the US.

## Methods

The Safety Program was conducted from December 1, 2017, to November 30, 2018, and data analysis was performed from March 1 to October 31, 2019. Any acute care facility (either an entire hospital or units within a hospital) in the US was eligible to participate, with a predetermined maximum enrollment of 500 sites. Each site identified a medical and pharmacy ASP director. All clinical staff were encouraged to be involved in the Safety Program, including physicians, pharmacists, physician assistants, nurse practitioners, and nurses, and each individual was provided unique credentials to access content. Continuing education credits were provided. This study followed the Standards for Quality Improvement Reporting Excellence (SQUIRE) reporting guideline.^5^

The Safety Program content described herein is now publicly available as part of the AHRQ Toolkit for Improving Antibiotic Use^6^ (Table 1). Seventeen webinars (each repeated 3 times and recorded for participants) occurred during the 12-month period. Two of us (P.D.T. and S.E.C.) led all webinars to enhance continuity across the span of the program and build ongoing relationships with participants. Webinars focusing on the best practices in the diagnosis and management of infectious diseases processes for which antibiotics are frequently prescribed in the inpatient setting used the Four Moments of Antibiotic Decision Making framework (Box).^7^ This framework, created for the Safety Program, encourages clinicians to address time points when antibiotic plans should be reviewed, prompting decisions around obtaining appropriate cultures and initiating antibiotic therapy; discontinuing, narrowing, or transitioning from intravenous to oral antibiotic therapy; and selecting the safest yet most effective treatment duration.

**Table 1.** Components of the AHRQ Safety Program

Implementation element	Goal	Frequency	Intended audience
Antibiotic stewardship program implementation webinars	Provide strategies to develop or enhance antibiotic stewardship infrastructure, implement interventions, and measure program outcomes	Every 2 wk for the first 6 wk of the program^a^	Stewardship team
Science of antibiotic safety webinars	Provide information to frame antibiotic use as a patient safety issue, improve teamwork and communication around antibiotic decision-making, and identify antibiotic-associated harm and develop solutions to prevent it	Every 2 wk for the second 6 wk of the program^a^	Stewardship team and frontline clinicians
Infectious disease syndrome webinars	Provide guidance for best practices in the diagnosis and management of common infectious disease syndromes (asymptomatic bacteriuria, urinary tract infections, community-acquired and hospital-acquired respiratory tract conditions, cellulitis and soft tissue abscesses, diverticulitis and biliary tract infections, *Clostridioides difficile* infection, sepsis, and bacteremia) using the Four Moments of Antibiotic Decision-Making framework^7^	Monthly during mo 4-11 of the program^a^	Stewardship team and frontline clinicians
Sustainability webinar	Provide guidance for maintaining the stewardship work that occurred during the program, after the 1-y program is completed	Last month of the project^a^	Stewardship team
Narrated presentations	Provide detailed guidance on targeted topics (collaboration with the clinical microbiology laboratory, integrating nurses into stewardship activities, antibiotic allergies)	Available on the project website for independent access	Stewardship team and frontline clinicians
1-Page documents and accompanying user guides	Provide succinct guidance on the infectious disease syndromes covered in the webinars; editable to enter institution-specific antibiotic recommendations. Document could be used as (1) informational posters, (2) discussion points on clinical rounds, or (3) outline for developing local guidelines. Accompanying user guides include specific antibiotic information for children and adults and other pertinent information to include if developing local guidelines.	Available on the project website for independent access	Stewardship team and frontline clinicians
Team Antibiotic Review Form	Document to be completed by frontline prescribers in conjunction with antibiotic stewards for patients actively receiving antibiotics to facilitate discussions about appropriate antibiotic prescribing using the Four Moments framework	Sites required to complete 10 TARFs/mo during mo 4-11 of the project	Stewardship team and frontline clinicians
Four Moments of Antibiotic Decision-Making posters, pocket cards, and screen savers	Provide posters and pocket cards advertising the Four Moments framework (Box)^7^	Available on the project website for independent access	Stewardship team and frontline clinicians
Judicious antibiotic prescribing commitment posters	Display poster branded with local logos, photos, and signatures to indicate to patients and staff that the unit was committed to judicious antibiotic prescribing	Available on the project website for independent access	Stewardship team and frontline clinicians
Antibiotic time-out tool	Review tool for use during clinical rounds prompting a daily review of antibiotic therapy	Available on the project website for independent access	Frontline clinicians
Identifying antibiotic-associated adverse events form; learning from antibiotic-associated adverse events form	Tools to identify antibiotic safety concerns and to propose potential solutions to prevent future antibiotic-associated harm	Available on the project website for independent access	Stewardship team and frontline clinicians
Office hours	Provide a forum for participants to ask the project team questions about project logistics, implementation strategies, clinical management strategies, and to share local successes and challenges. Project email address and designated, external site-specific quality improvement expert are also available to all participants at each site	Twice monthly	Stewardship team and frontline clinicians

Abbreviation: AHRQ, Agency for Healthcare Research and Quality.

^a^ All webinars were recorded and had an associated slide set and detailed script material; could be accessed on the project website by all participants in the Safety Program at any time. Each webinar topic was presented 3 times to account for different time zones. All webinars led by the same individuals (P.D.T. and S.E.C.) to enhance continuity across the span of the Safety Program and to build ongoing relationships with participants.

Box. **The Four Moments of Antibiotic Decision-Making Framework**

**1. Make the Diagnosis**

Does my patient have an infection that requires antibiotics?

**2. Cultures and Empiric Therapy**

Have I ordered appropriate cultures before starting antibiotics? What empiric therapy should I initiate?

**3. Stop, Narrow, Change to Oral Antibiotics**

A day or more has passed. Can I stop antibiotics? Can I narrow therapy or change from intravenous to oral therapy?

**4. Duration**

What duration of antibiotic therapy is needed for my patient’s’ diagnosis?

Adapted from Tamma et al, 2019.^7^

Sites were requested to complete and upload 10 Team Antibiotic Review Forms (TARF) per month per participating unit to the Safety Program website beginning in March 2018 (eAppendix in the Supplement).^6^ The TARF used the Four Moments framework and was jointly completed by frontline prescribers and the ASP for patients actively receiving antibiotics to foster discussion surrounding appropriate antibiotic prescribing.

Participants had access to the Safety Program project team throughout the year via question and answer sessions after webinars, twice-monthly office hours led by the individuals who conducted the webinars (P.D.T. and S.E.C.), and access to a Safety Program email address for additional questions. An external quality improvement expert well-versed in large-scale implementation programs in hospital settings^8^ was assigned to each site by the Safety Program to lend additional assistance with implementing the Safety Program and interacted by telephone with each site at least monthly.

A gap analysis describing local ASP infrastructure was completed at the beginning and end of the Safety Program (eAppendix in the Supplement).^6^ Before the onset of the Safety Program, 2 of us (P.D.T. and S.E.C.) performed a systematic review to determine key components of successful ASPs (eAppendix in the Supplement). Four key components were identified: (1) active interventions before and after prescription of select antibiotics, (2) local guidelines available for treatment of urinary tract infections and community-acquired pneumonia, (3) physician and pharmacist ASP leads with dedicated salary support, and (4) quarterly tracking and reporting of antibiotic use.

The primary outcome of the Safety Program was unit-level, or hospital-level for critical access hospitals, antibiotic use data measured as days of antibiotic therapy (DOT) per 1000 patient days (PD). Monthly antibiotic use data were submitted for 50 intravenous or oral antibiotics from January 1 to December 31, 2018, beginning the month following the onset of the Safety Program (eAppendix in the Supplement). Quarterly hospital-onset *Clostridioides difficile* rates were submitted as *C difficile* laboratory-identified (LabID) events per 10 000 PD for the participating units as a proxy for *C difficile* infection.^9^ Individualized quarterly data reports were provided to each site to assess progress over the course of the Safety Program and compare similar units and hospital types (eAppendix in the Supplement).

Multiple steps were followed to ensure valid data collection. At the beginning of the Safety Program, an informational webinar provided detailed instructions regarding data collection. Sites unfamiliar with electronic data extraction were introduced to sites able to successfully navigate the same electronic health record system to access antibiotic use data. Sites without electronic medical record systems were instructed on accurate manual collection of data. A standardized template with detailed instructions was used to collect and upload data (eAppendix in the Supplement). The quality improvement expert assigned to each hospital assisted with troubleshooting data collection issues. Sites reporting antibiotic use rates significantly higher or lower than expected, suggesting an error in collection of the numerator or denominator, were requested to re-extract data.

### Statistical Analysis

The hospital served as the unit of analysis for the gap analysis and adherence to the 4 key successful ASP elements, with comparisons made using the χ^2^ test. The hospital unit served as the unit of analysis of antibiotic use data and *C difficile* LabID events.

Antibiotic use data were grouped into bimonthly intervals, enabling comparisons of each bimonthly interval (eg, March-April, May-June) with the January-February baseline. Changes in overall DOT per 1000 PD during the intervention based on select hospital and unit characteristics were also estimated.

To investigate whether there was an association between high engagement in the Safety Program and greater reduction in antibiotic use, we performed a stratified analysis comparing changes in antibiotic use between units that completed an average of 10 or more TARFs per month (ie, high engagement) with those that completed less than 10 TARFs per month. In addition, a subgroup analysis of changes in fluoroquinolone (ie, ciprofloxacin, levofloxacin, and moxifloxacin) DOT per 1000 PD from the beginning to the end of the Safety Program were compared, because a large focus of the Safety Program was reducing unnecessary fluoroquinolone use.

A linear mixed model with random hospital unit effects was used to assess changes in overall antibiotic use from baseline (January-February 2018) to the end of the Safety Program (November-December 2018), as well as from baseline to each bimonthly period. A generalized linear model with random hospital unit effects was used to estimate changes from quarter 1 (January 2018-March 2018) to each of the subsequent 3 quarters for hospital-onset *C difficile* LabID events, with the additional assumption that the number of events followed a Poisson distribution, given that *C difficile* LabID events are relatively rare. Incidence rate ratios described changes in *C difficile* infection rates over time.

Antibiotic use data from the Premier Healthcare Database, a database containing drug use information for approximately one-fourth of hospitalizations in the US, were analyzed to provide information on temporal antibiotic changes during the period of the Safety Program.^10^ Hospitals that provided days of antibiotic therapy per 1000 PD for all antibiotics included in the Safety Program and for all months from January to December 2018 were included in the Premier Healthcare Database cohort. Entropy balancing was used to ensure a similar distribution of academic and nonacademic hospitals as well as intensive care unit and general wards in the Premier Healthcare Database cohort in an attempt to mirror the Safety Program. Hospitals that participated in the Safety Program were excluded from the Premier Healthcare Database cohort. In total, 1711 units located in 614 hospitals in the Premier Healthcare Database were included in the analysis. Antibiotic use data from January-February to November-December and from baseline to each subsequent bimonthly period in the Premier Healthcare Database cohort were analyzed using an identical approach as antibiotic use data from Safety Program sites. The presumed residual heterogeneity of the Premier Healthcare Database cohort compared with the Safety Program cohort (eg, exclusion of critical access hospitals, unavailability of data on stewardship infrastructure), precluded direct comparisons between the Safety Program and Premier Healthcare Database cohort antibiotic use data. Data analyses were conducted using SAS, version 9.4 (SAS Institute Inc). Findings were considered significant at *P* < .05 with 2-sided testing.

## Results

### Safety Program Enrollment

Overall, 437 hospitals enrolled in the AHRQ Safety Program; of these, 402 (92%) hospitals, which included 476 units, remained in the Safety Program until its completion. The eFigure in the Supplement illustrates the distribution of enrolled hospitals across the US. The 402 hospitals included 28 (7%) academic medical centers, 122 (30%) midlevel teaching hospitals, 167 (42%) community hospitals, and 85 (21%) critical access hospitals (Table 2). This distribution translated to 165 hospitals (41%) with 100 or fewer beds, 136 (34%) hospitals with 101 to 299 beds, and 101 (25%) hospitals with 300 or more beds. A total of 261 (65%) hospitals were urban/suburban and 141 (35%) hospitals were rural. A total of 173 hospitals (43%) did not have access to infectious diseases specialists at the beginning of the Safety Program. At the start of the Safety Program, 8% of participating hospitals reported adherence to all 4 key ASP components. By the end of the Safety Program, adherence increased to 74% (*P* < .01).

**Table 2.** Baseline Characteristics of the 402 Hospitals Participating in the AHRQ Safety Program

Characteristic	No. (%)
Hospital type	
Academic medical center	28 (7)
Midlevel teaching hospital	122 (30)
Community hospital	167 (42)
Critical access hospital	85 (21)
Participating units (n = 476)	
Medical ward	94 (20)
Surgical ward	11 (2)
Medical-surgical ward	195 (41)
Intensive care units	165 (35)
Other	11 (2)
Hospital size, beds	
≥300	101 (25)
101-299	136 (34)
≤100	165 (41)
Facility location	
Urban or suburban	261 (65)
Rural location	141 (35)
Access to infectious diseases specialists	229 (57)
Interventions before and after prescription of antibiotics	84 (21)
Availability of local antibiotic guidelines for community-acquired pneumonia and urinary tract infections	88 (22)
Dedicated salary support for ASP physician and pharmacist lead	32 (8)
Quarterly collection and reporting of antibiotic use	92 (23)
US Department of Health and Human Services region	
Connecticut, Maine, Massachusetts, New Hampshire, Rhode Island, and Vermont	25 (6)
New Jersey, New York, Puerto Rico, and the Virgin Islands	24 (6)
Delaware, District of Columbia, Maryland, Pennsylvania, Virginia, and West Virginia	31 (8)
Alabama, Florida, Georgia, Kentucky, Mississippi, North Carolina, South Carolina, and Tennessee	56 (14)
Illinois, Indiana, Michigan, Minnesota, Ohio, and Wisconsin	62 (15)
Arkansas, Louisiana, New Mexico, Oklahoma, and Texas	80 (20)
Iowa, Kansas, Missouri, and Nebraska	45 (11)
Colorado, Montana, North Dakota, South Dakota, Utah, and Wyoming	29 (7)
Arizona, California, Hawaii, Nevada, and USAPI (American Samoa, Commonwealth of the Northern Mariana Islands, Federated States of Micronesia, Guam, Marshall Islands, and Republic of Palau)	29 (7)
Alaska, Idaho, Oregon, and Washington	21 (5)

Abbreviation: AHRQ, Agency for Healthcare Research and Quality.

The AHRQ Safety Program website had 11 650 unique users. There was a median of 561 attendees per webinar (range, 467-793); on average, 511 participants (91%) described the content as excellent and 45 participants (8%) described the content as very good, as reported through blinded surveys at the conclusion of each webinar. There was an average of 470 downloads per item from the Safety Program website (eTable in the Supplement). The Safety Program Help Desk received 3889 initial email inquiries from participants.

### Unit-Level Antibiotic Use Data

Comparing January-February with November-December 2018, antibiotic use decreased from 900.7 to 870.4 DOT per 1000 PD (−30.3 DOTs; 95% CI, −52.6 to −8.0 DOT; *P* = .008). Evaluating bimonthly antibiotic use, the largest decrease occurred between January-February and March-April (Figure). This decrease was sustained for the remainder of the cohort. Point estimates of antibiotic use changes for specific hospital and unit types also indicated reductions in antibiotic use (Table 3). Fluoroquinolone use decreased from 105.0 to 84.6 DOT per 1000 PD across all units between January-February and November-December (−20.4 DOT; 95% CI, −25.4 to −15.5 DOT; *P* = .009).

**Figure.**
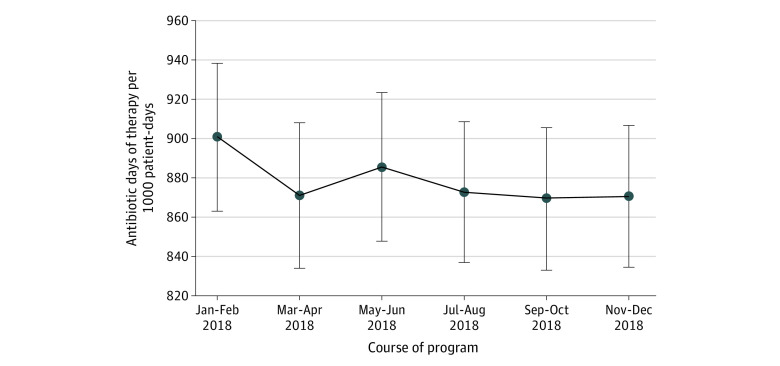
Changes in Overall Antibiotic Days of Therapy per 1000 Patient-Days Over the Course of the 1-Year Agency for Healthcare Research and Quality Safety Program for Improving Antibiotic Use

**Table 3.** Changes in Antibiotic Use Comparing November-December 2018 With January-February 2018 Across 402 Hospitals Participating in the AHRQ Safety Program^a^

Variable	No.	DOT per 1000 patient-days		Mean difference (95% CI)	*P* value
		Jan-Feb	Nov-Dec		
Use in all hospitals					
All antibiotics	402	900.7	870.4	−30.3 (−52.6 to −8.0)	.008
Fluoroquinolones	402	105.0	84.6	−20.4 (−25.4 to −15.5)	.009
Antibiotic use					
In academic medical centers	28	854.4	808.4	−46.0 (−101.8 to 9.8)	.11
In nonacademic medical centers	374	906.8	878.7	−28.1 (−52.2 to −3.9)	.02
Antibiotic use in hospitals					
With ≤100 beds	165	888.2	863.7	−24.5 (−65.3 to 16.3)	.24
With 101-299 beds	136	875.6	850.9	−24.7 (−61.8 to 12.4)	.19
With ≥300 beds	101	939.6	896.8	−42.8 (−78.0 to −7.6)	.02
Antibiotic use					
In general wards	311	851.4	828.5	−22.9 (−48.6 to 2.8)	.08
In intensive care units	165	1001.9	962.7	−39.2 (−82.9 to 4.6)	.08
Antibiotic use in hospitals					
Adherent to all 4 key components of antibiotic stewardship at baseline	32	876.5	826.9	−49.6 (−106.3 to 7.1)	.09
Not adherent to all 4 key components of antibiotic stewardship at baseline	370	903.9	875.9	−28.0 (−52.0 to −3.9)	.02

Abbreviations: AHRQ, Agency for Healthcare Research and Quality; DOT, days of antibiotic therapy.

^a^ Data analysis occurred using linear mixed models with random hospital unit effects.

Changes in antibiotic use between high engagement units and units with low to average engagement were compared. For 200 units that completed an average of less than 10 TARFs per month, antibiotic use decreased from 861 to 845 DOT per 1000 PD between January-February and November-December 2018; −15.6 DOT (*P* = .55). For 276 units that completed an average of 10 or more TARFs per month, antibiotic use decreased from 912 to 877 DOT per 1000 PD; −34.2 DOT (*P* < .01), suggesting that units more actively engaged in the Safety Program were more likely to see a decrease in antibiotic use.

In contrast to the Safety Program, there were no significant reductions in antibiotic use observed in the Premier Healthcare Database cohort when comparing January-February with November-December 2018. Overall antibiotic DOT per 1000 PD decreased by −9.0 (95% CI, −44.4 to 26.4, *P* = .62) from 648 in January-February to 639 DOT per 1000 PD in November-December for the Premier Healthcare Database sample. Furthermore, when comparing January-February with March-April, the period of the largest decrease in antibiotic use in the Safety Program, overall antibiotic use in the Premier Healthcare Database cohort increased from 648 to 660 DOT per 1000 PD.

### *C difficile* LabID Events

The number of hospital-onset *C difficile* LabID events per 10 000 PD across the Safety Program cohort was 6.3 for quarter 1, 5.3 for quarter 2, 6.0 for quarter 3, and 5.1 for quarter 4 in the 2018 calendar year in the participating units. The incidence rate of hospital-onset *C difficile* LabID events decreased from quarter 1 to quarter 4 by 19.5% (95% CI, −33.5% to −2.4%, *P* = .03).

## Discussion

The AHRQ Safety Program for Improving Antibiotic Use is the largest hospital antibiotic stewardship quality improvement initiative in the US to date. It contributed to a decrease in overall antibiotic use and *C difficile* infection across 402 US hospitals, comparing the beginning and end of the 1-year program, with the greatest reductions in units highly engaged in the Safety Program. A large proportion of hospitals were underresourced. Forty-one percent of sites had 100 hospital beds or less, including 85 critical access hospitals, 43% did not have access to infectious diseases specialists, and 35% self-described as rural hospitals.

Observing meaningful changes after the establishment of an ASP takes time owing to delays with obtaining adequate acceptance of stewardship principles from other clinicians, developing local guidelines, and changing long-held beliefs surrounding antibiotics.^11,12^ Despite these barriers, we observed a decrease in antibiotic use in the initial year of the Safety Program. The Safety Program simultaneously provided comprehensive training to ASP leads as well as frontline clinicians and included live webinars as well as durable educational material, content to increase buy-in for stewardship principles (eg, commitment posters, computer desktop backgrounds, and pocket cards), and training on collating and feeding-back antibiotic use data, all of which expedited the establishment and augmentation of ASPs. Hospitals with nascent or no ASPs are encouraged to access the Toolkit Implementation Guide for Acute Care Antibiotic Stewardship Programs to emulate the approaches used in the Safety Program (eAppendix in the Supplement).^6^

We observed a significant reduction in fluoroquinolone use. Owing to a combination of their dosing convenience, efficacy, and ability to successfully penetrate a broad range of body sites, fluoroquinolones are frequently prescribed as first-line treatment agents, even when alternative agents with a more favorable adverse-event profile exist.^13,14,15,16^ There is a growing body of evidence describing adverse events associated with fluoroquinolone use, including prolonged QTc intervals, tendinitis and tendon rupture, aortic dissections, seizures, peripheral neuropathy, and *C difficile* infection.^17,18,19,20,21,22,23^ A study evaluating 549 acute care facilities found that hospitals that decreased fluoroquinolone use by at least 20% experienced an 8% decrease in *C difficile* infection rates.^22^ The Safety Program educated clinicians on adverse events associated with fluoroquinolones and on identifying alternative antibiotics for situations in which fluoroquinolones are commonly used.^6^ The significant reduction in fluoroquinolone use observed during the Safety Program may have contributed to the reduction in hospital-onset *C difficile* LabID events.

Although we would like to fully attribute the decrease in overall antibiotic use to the success of the Safety Program, there are alternative explanations, including the following: (1) the influence of seasonal changes, (2) expected secular variations, or (3) inaccuracies with submitted antibiotic use data, each described in further detail below. Because the Safety Program was a quality improvement initiative and not a research study, we limited data collection to information necessary to improve local antibiotic prescribing practices. We did not want to discourage resource-limited hospitals (eg, rural hospitals, critical access hospitals), traditionally excluded from large quality improvement initiatives, from participating by requesting data that would be overly onerous to obtain. As such, we did not collect antibiotic use data in the year before the Safety Program to serve as a control group.

Seasonal changes are an important determinant of antibiotic prescribing rates.^24,25,26,27,28,29^ More specifically, antibiotic use increases in the winter months. Suda and colleagues^24^ investigated antibiotic prescriptions in the US over a 5-year period and reported that they were almost 25% higher in winter months (defined as the first and last quarters of each year) compared with summer months. Similarly, an evaluation of antibiotic consumption in British Columbia over a 4-year period revealed that antibiotic use during quarters 1 and 4 were 22% greater than in quarters 2 and 3.^29^ Although seasonal changes may have influenced the high antibiotic prescribing rate observed in January-February in the Safety Program, seasonality was unlikely to have factored prominently into our findings, given that both the beginning (ie, January-February) and end (ie, November-December) of the Safety Program occurred in winter months. Moreover, antibiotic use data from the Premier Healthcare Database indicate that antibiotic use was similar in January-February and November-December 2018. In addition, we do not have evidence from the literature suggesting that antibiotic use in January-February is generally higher than November-December.^28,30,31,32,33^

Decreased antibiotic use during the course of the Safety Program may have reflected secular changes. With a growing understanding of the need for judicious antibiotic prescribing due to public health campaigns,^34^ an upsurge in the lay and scientific literature focusing on antibiotic-associated harm,^35,36,37,38,39,40,41^ an enhanced evidence base supporting shorter durations of therapy than historically prescribed,^42,43^ and recommendations for minimizing prescribing for common indications, such as perioperative prophylaxis^44^ or asymptomatic bacteriuria,^45^ antibiotic use may have decreased over time in participating hospitals independent of the Safety Program.

To explore whether Safety Program antibiotic use changes represented secular trends, we obtained 2018 data from the Premier Healthcare Database. In contrast to the Safety Program, no significant reductions in antibiotic use were observed from January-February to November-December in the Premier Healthcare Database cohort. Moreover, no significant reductions in antibiotic use were observed between January-February and March-April in the Premier Healthcare Database cohort, the period with the sharpest decline in antibiotic use in the Safety Program cohort. Comparisons between the Safety Program and the Premier Healthcare Database cohort should be interpreted cautiously because some important information was not available for the hospitals contributing data to the Premier Healthcare Database cohort. Important missing information included antibiotic stewardship infrastructure, ongoing quality improvement activities, and access to infectious diseases specialists. Furthermore, critical access hospitals were not represented in the Premier Healthcare Database cohort. These limitations notwithstanding, antibiotic use data from the Premier Healthcare Database cohort do not suggest temporal trends indicating a reduction in antibiotic use over the 2018 calendar year.

We explored the literature to understand whether antibiotic use has followed a decreasing trend over the past several years. Baggs and colleagues^46^ evaluated antibiotic use across more than 300 US hospitals using the Truven Health MarketScan Hospital Drug Database from 2006 to 2012 and did not observe decreases in overall antibiotic use during this 7-year period. Goodman and colleagues^47^ performed an almost identical analytic approach as Baggs et al, using the Premier Healthcare Database from 2016 to 2017 to investigate antibiotic use data across 576 US hospitals and found total antibiotic consumption was not substantially different from antibiotic use data published almost a decade earlier.^46^ These data do not provide strong support for the narrative that antibiotic use was expected to naturally decline over the 2018 calendar year. In addition, with the recommendation of The Surviving Sepsis Campaign Bundle: 2018 Update that all elements of the Severe Sepsis/Septic Shock Early Management Bundle be initiated within 1 hour of emergency department presentation, it is plausible that antibiotic use would be anticipated to gradually increase during 2018.^48^

### Limitations

The study has limitations. In addition to the aforementioned discussion of the lack of a control group and seasonality concerns, we cannot discount the possibility of inaccuracies with antibiotic data collection by participating sites. Our lack of access to protected health information precluded our ability to ensure the integrity of antibiotic use data submitted from hospitals or an evaluation of appropriate antibiotic use. Nonetheless, rigorous steps were followed to maximize the likelihood of valid data submission as previously described.

## Conclusions

The AHRQ Safety Program for Improving Antibiotic Use was associated with a significant decrease in overall antibiotic use and hospital-onset *C. difficile* infection rates across 402 US hospitals. The Centers for Medicare & Medicaid Services required that hospitals—including critical access hospitals—implement ASP by March 30, 2020, as a condition of participation.^49^ Hospitals that had not yet established ASPs likely included large numbers of resource-limited hospitals, similar to many that participated in the Safety Program. The Safety Program was able to demonstrate that establishing ASPs and training frontline clinicians may improve antibiotic use, even in hospitals without infectious diseases–trained physicians or pharmacists. In fact, we were able to train clinicians—often staff pharmacists—to become stewardship leaders in their facilities. Because all content from the Safety Program is publicly available,^6^ use of Safety Program resources provides opportunities for hospitals across the US seeking to improve antibiotic use by establishing or strengthening existing ASPs and teaching frontline clinicians to become self-stewards of their antibiotic use.

## Notes

Supplement. **eAppendix.** Detailed Methods **eReferences** **eTable.** Metrics of the Top 30 Downloaded AHRQ Safety Program Content From December 2017 to November 2018 **eFigure.** Distribution of 402 Hospitals Across the United States Enrolled in the AHRQ Safety Program; Color Gradients Represent Number of Sites Enrolled per State

## References

[1] Tamma PD, Cosgrove SE. Antimicrobial stewardship. Infect Dis Clin North Am. 2011;25(1):245-260. doi:10.1016/j.idc.2010.11.011 21316003

[2] O’Leary EN, van Santen KL, Webb AK, Pollock DA, Edwards JR, Srinivasan A. Uptake of antibiotic stewardship programs in US acute care hospitals: findings from the 2015 National Healthcare Safety Network Annual Hospital Survey. Clin Infect Dis. 2017;65(10):1748-1750. doi:10.1093/cid/cix651 29020178

[3] Barlam TF, Cosgrove SE, Abbo LM, . Implementing an antibiotic stewardship program: guidelines by the Infectious Diseases Society of America and the Society for Healthcare Epidemiology of America. Clin Infect Dis. 2016;62(10):e51-e77. doi:10.1093/cid/ciw118 27080992 PMC5006285

[4] Pronovost PJ, Berenholtz SM, Goeschel C, . Improving patient safety in intensive care units in Michigan. J Crit Care. 2008;23(2):207-221. doi:10.1016/j.jcrc.2007.09.002 18538214

[5] Revised Standards for Quality Improvement Reporting Excellence. SQUIRE 2.0. Accessed December 31st, 2020. http://www.squire-statement.org/index.cfm?fuseaction=Page.ViewPage&PageID=471

[6] Agency for Healthcare Research and Quality. Antibiotic stewardship toolkits. Accessed January 23, 2021. www.ahrq.gov/antibiotic-use/index.html

[7] Tamma PD, Miller MA, Cosgrove SE. Rethinking how antibiotics are prescribed: incorporating the 4 moments of antibiotic decision making into clinical practice. JAMA. 2019;321(2):139-140. doi:10.1001/jama.2018.19509 30589917

[8] Quality Improvement Organizations. About QIN-QIOs. Accessed February 10th, 2020. http://www.qioprogram.org/about/why-cms-has-qios

[9] The Centers for Disease Control and Prevention. National Healthcare Safety Network. Multidrug-resistant organism & Clostridioides difficile infection (MDROS & CDI). Accessed February 12, 2020. https://www.cdc.gov/nhsn/PDFs/pscManual/12pscMDRO_CDADcurrent.pdf

[10] Premier Applied Sciences. Premier healthcare database: data that informs and performs. Published March 2, 2020. Accessed December 15, 2020. https://products.premierinc.com/downloads/PremierHealthcareDatabaseWhitepaper.pdf

[11] Fabre V, Cosgrove S. Antimicrobial stewardship: it takes a village. Jt Comm J Qual Patient Saf. 2019;45(9):587-588. 31331859 10.1016/j.jcjq.2019.06.002

[12] Logan AY, Williamson JE, Reinke EK, Jarrett SW, Boger MS, Davidson LE. Establishing an antimicrobial stewardship collaborative across a large, diverse health care system. Jt Comm J Qual Patient Saf. 2019;45(9):591-599. doi:10.1016/j.jcjq.2019.03.002 31054876

[13] Shapiro DJ, Hicks LA, Pavia AT, Hersh AL. Antibiotic prescribing for adults in ambulatory care in the USA, 2007-09. J Antimicrob Chemother. 2014;69(1):234-240. doi:10.1093/jac/dkt301 23887867

[14] Thompson ND, Penna A, Eure TR, . Epidemiology of antibiotic use for urinary tract infection in nursing home residents. J Am Med Dir Assoc. 2020;21(1):91-96. doi:10.1016/j.jamda.2019.11.009 31822391

[15] Van Boeckel TP, Gandra S, Ashok A, . Global antibiotic consumption 2000 to 2010: an analysis of national pharmaceutical sales data. Lancet Infect Dis. 2014;14(8):742-750. doi:10.1016/S1473-3099(14)70780-7 25022435

[16] Olesen SW, Barnett ML, MacFadden DR, Lipsitch M, Grad YH. Trends in outpatient antibiotic use and prescribing practice among US older adults, 2011-15: observational study. BMJ. 2018;362:k3155. doi:10.1136/bmj.k3155 30054353 PMC6062849

[17] European Medicines Agency. Fluoroquinolone and quinolone antibiotics: PRAC recommends restrictions on use New restrictions follow review of disabling and potentially long-lasting side effects. Published 2018. Accessed February 12, 2020. https://www.ema.europa.eu/en/medicines/human/referrals/quinolone-fluoroquinolone-containing-medicinal-products

[18] FDA updates warnings for fluoroquinolone antibiotics on risks of mental health and low blood sugar adverse reactions. Published July 10, 2018. Accessed January 24, 2021. www.fda.gov/news-events/press-announcements/fda-updates-warnings-fluoroquinolone-antibiotics-risks-mental-health-and-low-blood-sugar-adverse

[19] FDA warns about increased risk of ruptures or tears in the aorta blood vessel with fluoroquinolone antibiotics in certain patients. Published May 10, 2017. Accessed January 24, 2021. https://www.fda.gov/drugs/drug-safety-and-availability/fda-warns-about-increased-risk-ruptures-or-tears-aorta-blood-vessel-fluoroquinolone-antibiotics

[20] Tanne JH. FDA adds “black box” warning label to fluoroquinolone antibiotics. BMJ. 2008;337:a816. doi:10.1136/bmj.a816 18632714 PMC2483892

[21] Brown KA, Khanafer N, Daneman N, Fisman DN. Meta-analysis of antibiotics and the risk of community-associated *Clostridium difficile* infection. Antimicrob Agents Chemother. 2013;57(5):2326-2332. doi:10.1128/AAC.02176-12 23478961 PMC3632900

[22] Kazakova SV, Baggs J, McDonald LC, . Association between antibiotic use and hospital-onset *Clostridioides difficile* infection in US acute care hospitals, 2006-2012: an ecologic analysis. Clin Infect Dis. 2020;70(1):11-18. doi:10.1093/cid/ciz169 30820545

[23] Pépin J, Saheb N, Coulombe MA, . Emergence of fluoroquinolones as the predominant risk factor for *Clostridium difficile*–associated diarrhea: a cohort study during an epidemic in Quebec. Clin Infect Dis. 2005;41(9):1254-1260. doi:10.1086/496986 16206099

[24] Suda KJ, Hicks LA, Roberts RM, Hunkler RJ, Taylor TH. Trends and seasonal variation in outpatient antibiotic prescription rates in the United States, 2006 to 2010. Antimicrob Agents Chemother. 2014;58(5):2763-2766. doi:10.1128/AAC.02239-13 24590486 PMC3993241

[25] Sun L, Klein EY, Laxminarayan R. Seasonality and temporal correlation between community antibiotic use and resistance in the United States. Clin Infect Dis. 2012;55(5):687-694. doi:10.1093/cid/cis509 22752512

[26] Stenehjem E, Hersh AL, Sheng X, . Antibiotic use in small community hospitals. Clin Infect Dis. 2016;63(10):1273-1280. doi:10.1093/cid/ciw588 27694483

[27] Durkin MJ, Jafarzadeh SR, Hsueh K, . Outpatient antibiotic prescription trends in the United States: a national cohort study. Infect Control Hosp Epidemiol. 2018;39(5):584-589. doi:10.1017/ice.2018.26 29485018 PMC7967296

[28] Palmay L, Elligsen M, Walker SA, . Hospital-wide rollout of antimicrobial stewardship: a stepped-wedge randomized trial. Clin Infect Dis. 2014;59(6):867-874. doi:10.1093/cid/ciu445 24928294

[29] Patrick DM, Marra F, Hutchinson J, Monnet DL, Ng H, Bowie WR. Per capita antibiotic consumption: how does a North American jurisdiction compare with Europe? Clin Infect Dis. 2004;39(1):11-17. doi:10.1086/420825 15206046

[30] Newland JG, Stach LM, De Lurgio SA, . Impact of a prospective-audit-with-feedback antimicrobial stewardship program at a children’s hospital. J Pediatric Infect Dis Soc. 2012;1(3):179-186. doi:10.1093/jpids/pis054 26619405

[31] Jenkins TC, Knepper BC, Shihadeh K, . Long-term outcomes of an antimicrobial stewardship program implemented in a hospital with low baseline antibiotic use. Infect Control Hosp Epidemiol. 2015;36(6):664-672. doi:10.1017/ice.2015.41 25740560 PMC4836835

[32] Mehta JM, Haynes K, Wileyto EP, ; Centers for Disease Control and Prevention Epicenter Program. Comparison of prior authorization and prospective audit with feedback for antimicrobial stewardship. Infect Control Hosp Epidemiol. 2014;35(9):1092-1099. doi:10.1086/677624 25111916 PMC4198070

[33] Lee TC, Frenette C, Jayaraman D, Green L, Pilote L. Antibiotic self-stewardship: trainee-led structured antibiotic time-outs to improve antimicrobial use. Ann Intern Med. 2014;161(10)(suppl):S53-S58. doi:10.7326/M13-3016 25402404

[34] Centers for Disease Control and Prevention. Antibiotic prescribing and use in doctor’s offices. Accessed February 12, 2020. www.cdc.gov/antibiotic-use/community/materials-references/index.html

[35] Vox. The post-antibiotic era is here. Published November 14, 2019. Accessed February 12, 2020. www.vox.com/future-perfect/2019/11/14/20963824/drug-resistance-antibiotics-cdc-report

[36] The New York Times. We will miss antibiotics when they’re gone. Published January 18, 2017. Accessed February 12, 2020. https://www.nytimes.com/2017/01/18/opinion/how-to-avoid-a-post-antibiotic-world.html

[37] Boucher HW, Bakken JS, Murray BE. The United Nations and the urgent need for coordinated global action in the fight against antimicrobial resistance. Ann Intern Med. 2016;165(11):812-813. doi:10.7326/M16-2079 27653291

[38] Boucher HW, Murray BE, Powderly WG. Proposed US funding cuts threaten progress on antimicrobial resistance. Ann Intern Med. 2017;167(10):738-739. doi:10.7326/M17-1678 28869976

[39] Luepke KH, Suda KJ, Boucher H, . Past, present, and future of antibacterial economics: increasing bacterial resistance, limited antibiotic pipeline, and societal implications. Pharmacotherapy. 2017;37(1):71-84. doi:10.1002/phar.1868 27859453

[40] Talbot GH, Jezek A, Murray BE, ; Infectious Diseases Society of America. The Infectious Diseases Society of America’s 10 × ’20 initiative (10 new systemic antibacterial agents US Food and Drug Administration approved by 2020): is 20 × ’20 a possibility? Clin Infect Dis. 2019;69(1):1-11. doi:10.1093/cid/ciz089 30715222

[41] Tamma PD, Avdic E, Li DX, Dzintars K, Cosgrove SE. Association of adverse events with antibiotic use in hospitalized patients. JAMA Intern Med. 2017;177(9):1308-1315. doi:10.1001/jamainternmed.2017.1938 28604925 PMC5710569

[42] Spellberg B, Rice LB. Duration of antibiotic therapy: shorter is better. Ann Intern Med. 2019;171(3):210-211. doi:10.7326/M19-1509 31284302 PMC6736742

[43] Wald-Dickler N, Spellberg B. Short-course antibiotic therapy-replacing Constantine units with “shorter is better”. Clin Infect Dis. 2019;69(9):1476-1479. doi:10.1093/cid/ciy1134 30615129 PMC6792080

[44] Bratzler DW, Dellinger EP, Olsen KM, ; American Society of Health-System Pharmacists (ASHP); Infectious Diseases Society of America (IDSA); Surgical Infection Society (SIS); Society for Healthcare Epidemiology of America (SHEA). Clinical practice guidelines for antimicrobial prophylaxis in surgery. Surg Infect (Larchmt). 2013;14(1):73-156. doi:10.1089/sur.2013.9999 23461695

[45] Nicolle LE. Updated guidelines for screening for asymptomatic bacteriuria. JAMA. 2019;322(12):1152-1154. doi:10.1001/jama.2019.11640 31550011

[46] Baggs J, Fridkin SK, Pollack LA, Srinivasan A, Jernigan JA. Estimating national trends in inpatient antibiotic use among US hospitals from 2006 to 2012. JAMA Intern Med. 2016;176(11):1639-1648. doi:10.1001/jamainternmed.2016.5651 27653796 PMC10863902

[47] Goodman KE, Cosgrove SE, Pineles L, . Significant regional differences in antibiotic use across 576 US hospitals and 11,701,326 million adult admissions, 2016-2017. Clin Infect Dis. 2020;ciaa570. Published online May 18, 2020. doi:10.1093/cid/ciaa570 32421195 PMC8282314

[48] Levy MM, Evans LE, Rhodes A. The Surviving Sepsis Campaign Bundle: 2018 Update. Crit Care Med. 2018;46(6):997-1000. doi:10.1097/CCM.0000000000003119 29767636

[49] Department of Health and Human Services. Centers for Medicare & Medicaid Services. Medicare and Medicaid programs; regulatory provisions to promote program efficiency, transparency, and burden reduction; fire safety requirements for certain dialysis facilities; hospital and critical access hospital (CAH) changes to promote innovation, flexibility, and improvement in patient care. Published September 30, 2019. Accessed February 24, 2020. https://www.govinfo.gov/content/pkg/FR-2019-09-30/pdf/2019-20736.pdf

