# Supplementary material for: Association of a Safety Program for Improving Antibiotic Use With Antibiotic Use and Hospital-Onset Clostridioides difficile Infection Rates Among US Hospitals
Source: JAMA Netw Open. 2021 Feb 26;4(2):e210235. doi: 10.1001/jamanetworkopen.2021.0235 (PMC7910818; doi:10.1001/jamanetworkopen.2021.0235)
Supplement: Supplement. — eAppendix. Detailed Methods eReferences eTable. Metrics of the Top 30 Downloaded AHRQ Safety Program Content From December 2017 to November 2018 eFigure. Distribution of 402 Hospitals Across the United States Enrolled in the AHRQ Safety Program; Color Gradients Represent Number of Sites Enrolled per State [file jamanetwopen-e210235-s001.pdf]

Supplementary Online Content

Tamma PD, Miller MA, Dullabh P, et al. Association of a safety program for improving antibiotic use with antibiotic use and hospital-onset *Clostridioides difficile* infection rates among US hospitals. *JAMA Netw Open*. 2021;4(2):e210235. doi:10.1001/jamanetworkopen.2021.0235

eAppendix. Detailed Methods

eReferences

eTable. Metrics of the Top 30 Downloaded AHRQ Safety Program Content From December 2017 to November 2018

eFigure. Distribution of 402 Hospitals Across the United States Enrolled in the AHRQ Safety Program; Color Gradients Represent Number of Sites Enrolled per State

This supplementary material has been provided by the authors to give readers additional information about their work.

© 2021 Tamma D et al. *JAMA Network Open.*

eAppendix. Detailed Methods

Systematic Review

Prior to the start of the formal one-year AHRQ Safety Program for Improving Antibiotic Use, PDT and SEC conducted a Systematic Review of the published literature to identify key components associated with successful antibiotic stewardship programs (ASPs). They first reviewed the evidence compiled in "Implementing an Antibiotic Stewardship Program: Guidelines by the Infectious Diseases Society of America and the Society for Healthcare Epidemiology of America[1]." These guidelines were developed by an expert panel using the GRADE methodology, a rigorous systematic approach to reviewing literature[2], that included a PubMed search of articles in the English language published between 1946 and July 2013. To ensure that the most current body of evidence was used to guide the AHRQ Safety Program and to reduce redundancy between the systematic review and the previously published guidelines, an additional PubMed search utilizing identical search parameters to the prior search was conducted[1], but limited to August 2013 to October 2018. The search yielded 1,747 results, of which 64 studies were selected for full text review. Articles were excluded if they described ASPs or interventions outside of the acute care setting. Four key components of successful ASPs in the acute care setting that were identified including: (1) interventions before and after prescription of select antibiotics, (2) availability of local guidelines at a minimum for urinary tract infections and community-acquired pneumonia, (3) physician and pharmacist ASP leads with dedicated salary support, and (4) quarterly tracking and reporting of antibiotic use. These components are in large part consistent with the Centers for Disease Control and Prevention Core Elements of Antibiotic Stewardship Programs[3]. The importance of each component was underscored throughout the course of the Safety Program. Details supporting these components are provided below. Compliance with the four key components were compared for the 402 participating sites at the beginning (January-February 2018) and end (November-December 2018) of the

© 2021 Tamma D et al. *JAMA Network Open.*

Safety Program based on self-reporting provided through the gap analysis, exit interviews, and monthly qualitative data reported to the quality improvement expert assigned to each site.

Interventions before and after prescription of select antibiotics. Active interventions both before antibiotics are prescribed (prior-approval) and after antibiotics are prescribed (e.g., post-prescription review with feedback, interventions focusing on intravenous to oral antibiotic step-down therapy, interventions targeting duration of therapy) appear necessary for ASPs to successfully reduce unnecessary antibiotic use[4-22]. Prior-approval generally consists of a phone call to the stewardship team for select anti-infectives justifying the use of the agent before administration to the patient. This approach can reduce unnecessary antibiotic initiation, optimize the selection of empiric antibiotics, provide information on optimal diagnostic tests, and encourage infectious diseases consultations when necessary. Drawbacks of this approach include its focus on specific restricted agents, potential to disrupt frontline clinician workflow, potential to delay antibiotic administration for sepsis, and an inability to address downstream antibiotic use such as intravenous to oral conversion or duration of therapy. Post-prescription review with feedback generally occurs 48 to 72 hours after antibiotics are initiated, when more clinical and microbiological data are available to make recommendations. Advantages to this approach include greater flexibility in the timing of interventions and the ability to address targeted therapy decisions. However, post-prescription review generally does not impact the first few days of antibiotic therapy, which often constitute a large portion of inpatient antibiotic use. A hybrid approach including a component of prior approval and post-prescription review is most impactful.

Development of local guidelines. The development and dissemination of institutional guidelines for diagnosing and treating common infectious syndromes is a key component of successful ASPs[23-28]. Guidelines provide evidence-based and standardized diagnostic and treatment recommendations based on local epidemiology. Guidelines should discuss appropriate clinical criteria suggestive of bacterial infections, diagnostic testing, specific empiric and targeted therapy (including dosing and options for

© 2021 Tamma D et al. *JAMA Network Open.*

severe drug allergies), and appropriate durations of therapy. Ideally, inpatient antibiotic guidelines should be developed to target common indications for antibiotic use such as asymptomatic bacteriuria/urinary tract infections, community-acquired pneumonia, hospital and ventilator-associated pneumonia, skin and soft tissue infections, intra-abdominal infections, and sepsis[29] – which were all included in the Safety Program content[30]. However, at a minimum, as asymptomatic bacteriuria/urinary tract infections and community-acquired pneumonia constitute a significant portion of inpatient antibiotic prescribing[29], guidelines for these conditions should be developed and disseminated locally.

Physician and Pharmacist Leads. ASPs are most successful when both a physician and pharmacist lead are identified, both with dedicated salary support to ensure adequate time to perform daily stewardship functions[24,31-34]. Although the physician and pharmacist lead are encouraged to develop ASP goals together, unique roles for the physician leader may include underscoring to other clinicians how the overarching goals of the stewardship program are to optimize patient outcomes while preventing patient harm and to engage hospital executive leadership. Pharmacists typically conduct the majority of stewardship interventions and often lead efforts to compile and validate antibiotic use data. The pharmacist also functions as a liaison to the pharmacy and therapeutics committee to encourage agreement between stewardship and pharmacy goals.

Quarterly tracking and reporting of antibiotic use. Periodically compiling relevant antibiotic use data informs interventions led by the ASP and enables the ASP to monitor progress over time[35-39]. These data should also be made available to clinicians and hospital administration.

© 2021 Tamma D et al. *JAMA Network Open.*

eReferences

1. Barlam TF, Cosgrove SE, Abbo LM, et al. Executive Summary: Implementing an Antibiotic Stewardship Program: Guidelines by the Infectious Diseases Society of America and the Society for Healthcare Epidemiology of America. *Clin Infect Dis.* 2016;62(10):1197-1202.
2. Guyatt GH, Oxman AD, Vist GE, et al. GRADE: an emerging consensus on rating quality of evidence and strength of recommendations. *BMJ.* 2008;336(7650):924-926.
3. The Centers for Disease Control and Prevention. https://www.cdc.gov/antibiotic-use/healthcare/implementation/core-elements.html. Accessed January 21, 2019.
4. Tamma PD, Avdic E, Keenan JF, et al. What Is the More Effective Antibiotic Stewardship Intervention: Preprescription Authorization or Postprescription Review With Feedback? *Clin Infect Dis.* 2017;64(5):537-543.
5. Mehta JM, Haynes K, Wileyto EP, et al. Comparison of prior authorization and prospective audit with feedback for antimicrobial stewardship. *Infect Control Hosp Epidemiol.* 2014;35(9):1092-1099.
6. Anderson DJ, Watson S, Moehring RW, et al. Feasibility of Core Antimicrobial Stewardship Interventions in Community Hospitals. *JAMA Netw Open.* 2019;2(8):e199369.
7. Brink AJ, Messina AP, Feldman C, et al. Antimicrobial stewardship across 47 South African hospitals: an implementation study. *Lancet Infect Dis.* 2016;16(9):1017-1025.
8. Davey P, Marwick CA, Scott CL, et al. Interventions to improve antibiotic prescribing practices for hospital inpatients. *Cochrane Database Syst Rev.* 2017;2:CD003543.
9. Rzewuska M, Charani E, Clarkson JE, et al. Prioritizing research areas for antibiotic stewardship programmes in hospitals: a behavioural perspective consensus paper. *Clin Microbiol Infect.* 2019;25(2):163-168.
10. Ivers N, Jamtvedt G, Flottorp S, et al. Audit and feedback: effects on professional practice and healthcare outcomes. *Cochrane Database Syst Rev.* 2012(6):CD000259.
11. Cosgrove SE, Seo SK, Bolon MK, et al. Evaluation of postprescription review and feedback as a method of promoting rational antimicrobial use: a multicenter intervention. *Infect Control Hosp Epidemiol.* 2012;33(4):374-380.
12. Hurst AL, Child J, Pearce K, Palmer C, Todd JK, Parker SK. Handshake Stewardship: A Highly Effective Rounding-based Antimicrobial Optimization Service. *Pediatr Infect Dis J.* 2016;35(10):1104-1110.
13. Messacar K, Campbell K, Pearce K, et al. A Handshake From Antimicrobial Stewardship Opens Doors for Infectious Disease Consultations. *Clin Infect Dis.* 2017;64(10):1449-1452.
14. Hurst AL, Child J, Parker SK. Intervention and Acceptance Rates Support Handshake-Stewardship Strategy. *J Pediatric Infect Dis Soc.* 2019;8(2):162-165.
15. Bond SE, Chubaty AJ, Adhikari S, et al. Outcomes of multisite antimicrobial stewardship programme implementation with a shared clinical decision support system. *J Antimicrob Chemother.* 2017;72(7):2110-2118.
16. Hulscher ME, Grol RP, van der Meer JW. Antibiotic prescribing in hospitals: a social and behavioural scientific approach. *Lancet Infect Dis.* 2010;10(3):167-175.
17. Lorencatto F, Charani E, Sevdalis N, Tarrant C, Davey P. Driving sustainable change in antimicrobial prescribing practice: how can social and behavioural sciences help? *J Antimicrob Chemother.* 2018;73(10):2613-2624.
18. Stenehjem E, Hersh AL, Buckel WR, et al. Impact of Implementing Antibiotic Stewardship Programs in 15 Small Hospitals: A Cluster-Randomized Intervention. *Clin Infect Dis.* 2018;67(4):525-532.

© 2021 Tamma D et al. *JAMA Network Open.*

19. Agwu AL, Lee CK, Jain SK, et al. A World Wide Web-based antimicrobial stewardship program improves efficiency, communication, and user satisfaction and reduces cost in a tertiary care pediatric medical center. *Clin Infect Dis.* 2008;47(6):747-753.
20. Sick AC, Lehmann CU, Tamma PD, Lee CK, Agwu AL. Sustained savings from a longitudinal cost analysis of an internet-based preapproval antimicrobial stewardship program. *Infect Control Hosp Epidemiol.* 2013;34(6):573-580.
21. Scarpato SJ, Timko DR, Cluzet VC, et al. An Evaluation of Antibiotic Prescribing Practices Upon Hospital Discharge. *Infect Control Hosp Epidemiol.* 2017;38(3):353-355.
22. Yogo N, Haas MK, Knepper BC, Burman WJ, Mehler PS, Jenkins TC. Antibiotic prescribing at the transition from hospitalization to discharge: a target for antibiotic stewardship. *Infect Control Hosp Epidemiol.* 2015;36(4):474-478.
23. Schuts EC, Hulscher M, Mouton JW, et al. Current evidence on hospital antimicrobial stewardship objectives: a systematic review and meta-analysis. *Lancet Infect Dis.* 2016;16(7):847-856.
24. Tamma PD, Cosgrove SE. Antimicrobial stewardship. *Infect Dis Clin North Am.* 2011;25(1):245-260.
25. Avdic E, Cushinotto LA, Hughes AH, et al. Impact of an antimicrobial stewardship intervention on shortening the duration of therapy for community-acquired pneumonia. *Clin Infect Dis.* 2012;54(11):1581-1587.
26. Erickson RM, Tritle BJ, Spivak ES, Timbrook TT. Impact of an Antimicrobial Stewardship Bundle for Uncomplicated Gram-Negative Bacteremia. *Open Forum Infect Dis.* 2019;6(12):ofz490.
27. Yarbrough PM, Kukhareva PV, Spivak ES, Hopkins C, Kawamoto K. Evidence-based care pathway for cellulitis improves process, clinical, and cost outcomes. *J Hosp Med.* 2015;10(12):780-786.
28. Abbo LM, Hooton TM. Antimicrobial Stewardship and Urinary Tract Infections. *Antibiotics (Basel).* 2014;3(2):174-192.
29. Magill SS, Edwards JR, Beldavs ZG, et al. Prevalence of antimicrobial use in US acute care hospitals, May-September 2011. *JAMA.* 2014;312(14):1438-1446.
30. Agency for Healthcare Research and Quality. Antibiotic Stewardship Toolkits. www.ahrq.gov/antibiotic-use/index.html. Accessed May 13th, 2020. .
31. Doernberg SB, Abbo LM, Burdette SD, et al. Essential Resources and Strategies for Antibiotic Stewardship Programs in the Acute Care Setting. *Clin Infect Dis.* 2018;67(8):1168-1174.
32. Pulcini C, Morel CM, Tacconelli E, et al. Human resources estimates and funding for antibiotic stewardship teams are urgently needed. *Clin Microbiol Infect.* 2017;23(11):785-787.
33. Morris AM, Rennert-May E, Dalton B, et al. Rationale and development of a business case for antimicrobial stewardship programs in acute care hospital settings. *Antimicrob Resist Infect Control.* 2018;7:104.
34. Echevarria K, Groppi J, Kelly AA, Morreale AP, Neuhauser MM, Roselle GA. Development and application of an objective staffing calculator for antimicrobial stewardship programs in the Veterans Health Administration. *Am J Health Syst Pharm.* 2017;74(21):1785-1790.
35. Yu KC, Moisan E, Tartof SY, et al. Benchmarking Inpatient Antimicrobial Use: A Comparison of Risk-Adjusted Observed-to-Expected Ratios. *Clin Infect Dis.* 2018;67(11):1677-1685.
36. van Santen KL, Edwards JR, Webb AK, et al. The Standardized Antimicrobial Administration Ratio: A New Metric for Measuring and Comparing Antibiotic Use. *Clin Infect Dis.* 2018;67(2):179-185.
37. Fridkin SK, Srinivasan A. Implementing a strategy for monitoring inpatient antimicrobial use among hospitals in the United States. *Clin Infect Dis.* 2014;58(3):401-406.
38. Akpan MR, Ahmad R, Shebl NA, Ashiru-Oredope D. A Review of Quality Measures for Assessing the Impact of Antimicrobial Stewardship Programs in Hospitals. *Antibiotics (Basel).* 2016;5(1).

© 2021 Tamma D et al. *JAMA Network Open.*

39. Moehring RW, Anderson DJ, Cochran RL, et al. Expert Consensus on Metrics to Assess the Impact of Patient-Level Antimicrobial Stewardship Interventions in Acute-Care Settings. *Clin Infect Dis.* 2017;64(3):377-383.

© 2021 Tamma D et al. *JAMA Network Open.*

PREVENT HAIs Healthcare-Associated Infections

Gap Analysis for Antibiotic Stewardship Programs

Instructions: Complete this document to assess your antibiotic stewardship program (ASP) on an annual basis. The ASP areas addressed in this document are those that are discussed throughout the AHRQ Safety Program Toolkit.

Unmarked questions ask about basic structure and commonly utilized interventions. The questions labeled as Enhancing Components (+) address components that may enhance ASPs. Once your ASP is established, discuss whether implementation of the Enhancing Components might be of benefit to your program and what resources would be need to operationalize them.

For answers that are not non-yes/no or non-yes/no/not applicable, select all answers that apply.

Key: + = Enhancing Components

ASP Area	Answers	Comments
**Program Leadership**		
**Physician Leadership**		
**Has a physician leader been identified?**	☐ Yes<br>☐ No	
**Is the physician leader trained in infectious disease?**	☐ Yes<br>☐ No<br>☐ Not applicable (N/A)	
**What salary support [full-time equivalent (FTE) or amount/hour consulting] is received by the ASP physician leader?**		
**How much time does the ASP physician leader dedicate to ASP (% effort or hours/week)**		
**Is the ASP physician leader available to the ASP on a daily basis?**	☐ Yes<br>☐ No<br>☐ N/A	
**Pharmacist Leadership**		
**Has a pharmacist leader been identified?**	☐ Yes<br>☐ No	
**Is the pharmacist leader trained in infectious diseases?**	☐ Yes<br>☐ No<br>☐ N/A	
**Senior Executive Leadership**		

© 2021 Tamma D et al. *JAMA Network Open*.

ASP Area	Answers	Comments
**To whom does the ASP report?**		
**How often does ASP leadership meet with senior leadership?**	☐ Monthly<br>☐ Quarterly<br>☐ Annually<br>☐ Never<br>☐ Other:	
**Does senior leadership actively promote/support antibiotic stewardship (AS) prevention activities?**	☐ No<br>☐ Yes: ASP Committee member<br>☐ Yes: Provides adequate funding for ASP<br>☐ Yes: Provides funding for AS member training<br>☐ Yes: Promotes AS messages via newsletters, screen savers, etc.<br>☐ Yes: Provides back up to ASP if prescribers do not follow AS approaches<br>☐ Yes: Other:	
**Program Structure**		
**How many pharmacists staff the ASP?**	Number:<br>FTE for pharmacist leader:<br>FTE for other AS pharmacists:	
**Does ASP have access to a data analyst?**	Number:<br>Total FTE for AS effort:<br>☐ No access to a data analyst	
**+Does the ASP have a regular meeting with infection prevention to discuss issues relevant to both groups?**	☐ Yes<br>☐ No	
**Is a representative of the ASP involved in antibiotic formulary decisions?**	☐ Yes<br>☐ No	
**Is there a hospital wide ASP Committee that meets at least quarterly?**	☐ Yes<br>☐ No	
**Who chairs the ASP Committee?**		
**Who is on the ASP Committee?**<br><br>**Note: representatives from areas listed are suggestions for robust committee membership; not all committees will have all areas represented.**	☐ Senior executive<br>☐ Pharmacy department<br>☐ Front-line nurses<br>☐ Infectious diseases physicians<br>☐ Information technology<br>☐ Microbiology lab<br>☐ Infection control/hospital epidemiology<br>☐ Department of nursing<br>☐ Regulatory affairs	

© 2021 Tamma D et al. *JAMA Network Open.*

ASP Area	Answers	Comments
	☐ Department of quality improvement<br>☐ Department of patient safety<br>☐ Patient representative<br>☐ Other:<br>☐ N/A	
**What are the activities of the ASP Committee?**<br><br>**Note: activities listed are suggestions for committee activities; not all committees will perform all activities.**	☐ Review antibiotic use data and recommend improvement approaches<br>☐ Review the antibiogram and recommend improvement approaches<br>☐ Review *Clostridioides difficile* infection rates and recommend improvement approaches<br>☐ Perform proactive risk assessments to determine areas in which harm related to antibiotic prescribing could be avoided with intervention<br>☐ Review guidelines developed by the ASP<br>☐ Review materials for patient and healthcare worker education regarding optimal antibiotic prescribing<br>☐ Review ASP responses to antibiotic shortages<br>☐ Review approaches employed by the microbiology lab for reporting culture and susceptibility data<br>☐ Assure ASP and its procedures and policies meet relevant regulations<br>☐ N/A	
**Are minutes taken and distributed?**	☐ Yes<br>☐ No<br>☐ N/A	
**To what committee does the ASP Committee report?**		
**Does your ASP develop an annual plan outlining goals for the following year?**	☐ Yes<br>☐ No	
**Does your ASP perform an annual risk assessment to identify priorities?**	☐ Yes<br>☐ No	

© 2021 Tamma D et al. *JAMA Network Open.*

ASP Area	Answers	Comments
**Does your ASP have a binder or other document detailing how it is compliant with The Joint Commission Antimicrobial Stewardship Standard and/or Centers for Disease Control and Prevention's (CDC) *Core Elements of Hospital Antibiotic Stewardship Programs*?**	☐ Yes<br>☐ No	
**Guidelines**		
**Does your facility have facility-specific antibiotic treatment guidelines?**	☐ Yes<br>☐ No	
**Do your facility-specific guidelines cover the following syndromes?**	☐ Urinary tract infection<br>☐ Asymptomatic bacteriuria<br>☐ Community-acquired pneumonia<br>☐ Healthcare-acquired pneumonia<br>☐ Ventilator-associated pneumonia<br>☐ Intra-abdominal infections<br>☐ Skin and soft tissue infection<br>☐ Bacteremia<br>☐ Sepsis<br>☐ Surgical prophylaxis<br>☐ *Clostridioides difficile* infection<br>☐ Other:<br>☐ Other:<br>☐ Other:<br>☐ N/A	
**Who is involved in guideline development?**	☐ ASP members<br>☐ Non-AS infectious disease physicians<br>☐ Non-ASP pharmacists<br>☐ Front-line prescriber content experts<br>☐ Trainees (e.g., housestaff, fellows)<br>☐ Other:<br>☐ N/A	
**Do your guidelines provide recommendations on empiric therapy?**	☐ Yes<br>☐ No<br>☐ N/A	
**Do your guidelines provide recommendations on oral step-down therapy?**	☐ Yes<br>☐ No<br>☐ N/A	
**Do your guidelines provide recommendations on duration of therapy?**	☐ Yes<br>☐ No<br>☐ N/A	
**Are guidelines disseminated to prescribers at the point of care?**	☐ Yes<br>☐ No	

© 2021 Tamma D et al. *JAMA Network Open.*

ASP Area	Answers	Comments
	☐ N/A	
✚Do your facility guidelines provide recommendations about specific antibiotics?	☐ Yes<br>☐ No<br>☐ N/A	
✚Do your guidelines provide recommendations for diagnostic testing?	☐ Yes<br>☐ No<br>☐ N/A	
✚Do your facility guidelines provide recommendations about the interpretation of microbiology results (including rapid diagnostic tests)?	☐ Yes<br>☐ No<br>☐ N/A	
✚Are guidelines available in operating rooms detailing specific recommendations for surgical prophylaxis?	☐ Yes<br>☐ No<br>☐ N/A	

Interventions

Preauthorization and Post-prescription Review and Feedback

Instructions for this section: For each agent or class, indicate whether the ASP performs pre-authorization and/or post-prescription review and feedback, and the frequency of these interventions. Use the results from this section to determine if the type of intervention or antibiotics intervened upon are appropriate or	Antibiotic	Pre-authorization	Frequency	Post-prescription review and feedback	Frequency
	Cefazolin	☐ Yes<br>☐ No<br>☐ N/A	☐ Daily weekdays<br>☐ Daily 7 days<br>☐ 2–3 times/week<br>☐ Other:<br>☐ N/A	☐ Yes<br>☐ No<br>☐ N/A	☐ Daily weekdays<br>☐ Daily 7 days<br>☐ 2–3 times/week<br>☐ Other:<br>☐ N/A
	Ceftriaxone	☐ Yes<br>☐ No<br>☐ N/A	☐ Daily weekdays<br>☐ Daily 7 days<br>☐ 2–3 times/week<br>☐ Other:<br>☐ N/A	☐ Yes<br>☐ No<br>☐ N/A	☐ Daily weekdays<br>☐ Daily 7 days<br>☐ 2–3 times/week<br>☐ Other:<br>☐ N/A
	Cefepime	☐ Yes<br>☐ No<br>☐ N/A	☐ Daily weekdays<br>☐ Daily 7 days<br>☐ 2–3 times/week<br>☐ Other:<br>☐ N/A	☐ Yes<br>☐ No<br>☐ N/A	☐ Daily weekdays<br>☐ Daily 7 days<br>☐ 2–3 times/week<br>☐ Other:<br>☐ N/A

© 2021 Tamma D et al. *JAMA Network Open.*

Interventions					
**should be modified based on institutional data and other ASP concerns.**	Ceftaroline	☐ Yes<br>☐ No<br>☐ N/A	☐ Daily weekdays<br>☐ Daily 7 days<br>☐ 2–3 times/week<br>☐ Other:<br>☐ N/A	☐ Yes<br>☐ No<br>☐ N/A	☐ Daily weekdays<br>☐ Daily 7 days<br>☐ 2–3 times/week<br>☐ Other:<br>☐ N/A
	Piperacillin/ Tazobactam	☐ Yes<br>☐ No<br>☐ N/A	☐ Daily weekdays<br>☐ Daily 7 days<br>☐ 2–3 times/week<br>☐ Other:<br>☐ N/A	☐ Yes<br>☐ No<br>☐ N/A	☐ Daily weekdays<br>☐ Daily 7 days<br>☐ 2–3 times/week<br>☐ Other:<br>☐ N/A
	Aztreonam	☐ Yes<br>☐ No<br>☐ N/A	☐ Daily weekdays<br>☐ Daily 7 days<br>☐ 2–3 times/week<br>☐ Other:<br>☐ N/A	☐ Yes<br>☐ No<br>☐ N/A	☐ Daily weekdays<br>☐ Daily 7 days<br>☐ 2–3 times/week<br>☐ Other:<br>☐ N/A
	Carbapenems	☐ Yes<br>☐ No<br>☐ N/A	☐ Daily weekdays<br>☐ Daily 7 days<br>☐ 2–3 times/week<br>☐ Other:<br>☐ N/A	☐ Yes<br>☐ No<br>☐ N/A	☐ Daily weekdays<br>☐ Daily 7 days<br>☐ 2–3 times/week<br>☐ Other:<br>☐ N/A
	Fluoroquinolones	☐ Yes<br>☐ No<br>☐ N/A	☐ Daily weekdays<br>☐ Daily 7 days<br>☐ 2–3 times/week<br>☐ Other:<br>☐ N/A	☐ Yes<br>☐ No<br>☐ N/A	☐ Daily weekdays<br>☐ Daily 7 days<br>☐ 2–3 times/week<br>☐ Other:<br>☐ N/A
	Aminoglycosides	☐ Yes<br>☐ No<br>☐ N/A	☐ Daily weekdays<br>☐ Daily 7 days<br>☐ 2–3 times/week<br>☐ Other:<br>☐ N/A	☐ Yes<br>☐ No<br>☐ N/A	☐ Daily weekdays<br>☐ Daily 7 days<br>☐ 2–3 times/week<br>☐ Other:<br>☐ N/A
	Vancomycin IV	☐ Yes<br>☐ No	☐ Daily weekdays	☐ Yes<br>☐ No	☐ Daily weekdays<br>☐ Daily 7 days

© 2021 Tamma D et al. *JAMA Network Open.*

Interventions					
		☐ N/A	☐ Daily 7 days<br>☐ 2–3 times/week<br>☐ Other:<br>☐ N/A	☐ N/A	☐ 2–3 times/week<br>☐ Other:<br>☐ N/A
	Daptomycin	☐ Yes<br>☐ No<br>☐ N/A	☐ Daily weekdays<br>☐ Daily 7 days<br>☐ 2–3 times/week<br>☐ Other:<br>☐ N/A	☐ Yes<br>☐ No<br>☐ N/A	☐ Daily weekdays<br>☐ Daily 7 days<br>☐ 2–3 times/week<br>☐ Other:<br>☐ N/A
	Linezolid/ Tedizolid	☐ Yes<br>☐ No<br>☐ N/A	☐ Daily weekdays<br>☐ Daily 7 days<br>☐ 2–3 times/week<br>☐ Other:<br>☐ N/A	☐ Yes<br>☐ No<br>☐ N/A	☐ Daily weekdays<br>☐ Daily 7 days<br>☐ 2–3 times/week<br>☐ Other:<br>☐ N/A
	Ceftazidime/ Avibactam	☐ Yes<br>☐ No<br>☐ N/A	☐ Daily weekdays<br>☐ Daily 7 days<br>☐ 2–3 times/week<br>☐ Other:<br>☐ N/A	☐ Yes<br>☐ No<br>☐ N/A	☐ Daily weekdays<br>☐ Daily 7 days<br>☐ 2–3 times/week<br>☐ Other:<br>☐ N/A
	Ceftolozane/ Tazobactam	☐ Yes<br>☐ No<br>☐ N/A	☐ Daily weekdays<br>☐ Daily 7 days<br>☐ 2–3 times/week<br>☐ Other:<br>☐ N/A	☐ Yes<br>☐ No<br>☐ N/A	☐ Daily weekdays<br>☐ Daily 7 days<br>☐ 2–3 times/week<br>☐ Other:<br>☐ N/A
	Polymyxins	☐ Yes<br>☐ No<br>☐ N/A	☐ Daily weekdays<br>☐ Daily 7 days<br>☐ 2–3 times/week<br>☐ Other:<br>☐ N/A	☐ Yes<br>☐ No<br>☐ N/A	☐ Daily weekdays<br>☐ Daily 7 days<br>☐ 2–3 times/week<br>☐ Other:<br>☐ N/A
	Vancomycin PO	☐ Yes<br>☐ No<br>☐ N/A	☐ Daily weekdays<br>☐ Daily 7 days	☐ Yes<br>☐ No<br>☐ N/A	☐ Daily weekdays<br>☐ Daily 7 days<br>☐ 2–3 times/week

© 2021 Tamma D et al. *JAMA Network Open.*

Interventions					
			☐ 2–3 times/week<br>☐ Other:<br>☐ N/A		☐ Other:<br>☐ N/A
	Fidaxomicin	☐ Yes<br>☐ No<br>☐ N/A	☐ Daily weekdays<br>☐ Daily 7 days<br>☐ 2–3 times/week<br>☐ Other:<br>☐ N/A	☐ Yes<br>☐ No<br>☐ N/A	☐ Daily weekdays<br>☐ Daily 7 days<br>☐ 2–3 times/week<br>☐ Other:<br>☐ N/A

ASP Area	Answers	Comments
**Do you have software or another mechanism that identifies patients for daily review by the ASP?**	☐ Yes<br>☐ No	
**How do you make AS interventions?**	☐ Phone call to clinicians<br>☐ Text to clinicians<br>☐ Rounds with teams<br>☐ Note in medical record<br>☐ Other:<br>☐ N/A	
**Where do you document AS intervention?**	☐ No documentation<br>☐ Medical record: visible to clinicians<br>☐ Medical record: not visible to clinicians<br>☐ Database internal to ASP<br>☐ N/A	
**Does your program monitor adherence to AS interventions?**	☐ Yes<br>☐ No<br>☐ N/A	
**Other Interventions To Consider**		
**+Is there is a formal procedure for all clinicians to review the appropriateness of all antibiotics 48–72 hours after the initial orders (e.g., antibiotic time out)**	☐ No<br>☐ Yes<br>☐ Select units	
**+Is there a process for intravenous to oral conversion of antibiotics in the pharmacy?**	☐ Yes<br>☐ No	
**+Does your facility have order sets for any of the following indications?**	☐ Urinary tract infection<br>☐ Community-acquired pneumonia<br>☐ Healthcare-acquired pneumonia<br>☐ Ventilator-associated pneumonia<br>☐ Intra-abdominal infections	

© 2021 Tamma D et al. *JAMA Network Open.*

ASP Area	Answers	Comments
	☐ Skin and soft tissue infection<br>☐ Bacteremia<br>☐ Sepsis<br>☐ Surgical prophylaxis<br>☐ *Clostridioides difficile* infection<br>☐ Neutropenic fever<br>☐ Other:<br>☐ Other:<br>☐ Other:<br>☐ N/A	
**+Are there time-sensitive automatic stop orders for specified antibiotic prescriptions?**	☐ Yes<br>☐ No	
**+Are activities conducted by the ASP to target antibiotics commonly associated with *C. difficile* infection (e.g., fluoroquinolones, clindamycin, cephalosporins)**	☐ Yes<br>☐ No	
**+Are activities conducted by the ASP to reduce inappropriate treatment of asymptomatic bacteriuria?**	☐ Yes<br>☐ No	
**+Are activities conducted by the ASP to guide interpretation of procalcitonin results?**	☐ No procalcitonin testing<br>☐ Procalcitonin results not acted upon by ASP<br>☐ Yes: all patients<br>☐ Yes: select patients	
**+List interventions conducted by the ASP to improve antibiotic use outside of core interventions.**		
**Microbiology**		

© 2021 Tamma D et al. *JAMA Network Open.*

ASP Area	Answers	Comments
**Do you have an onsite microbiology lab?**	☐ Yes<br>☐ No	
**Does the ASP have a regular meeting with the microbiology lab to discuss issues relevant to both groups (e.g., interpretation of susceptibility tests, implementation of new diagnostic tests, etc.?)**	☐ Yes<br>☐ No	
**Does your microbiology laboratory develop an annual antibiogram?**	☐ Yes: Whole hospital<br>☐ Yes: Stratified by unit<br>☐ Yes: Urine isolates<br>☐ Yes: Blood isolates<br>☐ No	
**Does your microbiology lab follow Clinical and Laboratory Standards Institute (CLSI) guidelines for making the antibiogram?**	☐ Yes<br>☐ No<br>☐ N/A	
**Is the antibiogram disseminated to prescribers?**	☐ Yes<br>☐ No<br>☐ N/A	
**+Does your microbiology lab blind any culture or susceptibility results as a strategy to assist prescribers in selecting appropriate antibiotics?**	☐ Yes<br>☐ No	
**+Does your facility perform rapid diagnostics on blood?**	☐ Yes: Fungal organisms<br>☐ Yes: Gram-negative organisms<br>☐ Yes: Gram-positive organisms<br>☐ No	
**+Does your facility perform rapid diagnostics on other specimens?**	☐ Yes: Respiratory specimens for viruses<br>☐ Yes: Respiratory specimens for bacteria<br>☐ Yes: Cerebrospinal fluid<br>☐ Legionella urinary antigen<br>☐ *Streptococcus pneumoniae* urinary antigen<br>☐ Other:<br>☐ Other:<br>☐ No	

© 2021 Tamma D et al. *JAMA Network Open.*

ASP Area	Answers	Comments
+Does your ASP have any specific interventions to adjust antibiotic regimens based on rapid diagnostic results?	☐ Yes<br>☐ No	
**Data**		
**Antibiotic Use Metrics**		
Do you have access to antibiotic use data?	☐Yes<br>☐No	
If you have access to antibiotic use data, what type of data is it?	☐ Purchasing data<br>☐ Days of therapy/1,000 patient-days<br>☐ Days of therapy/1,000 days present (National Healthcare Safety Network denominator)<br>☐ Defined daily doses<br>☐ Other:<br>☐ N/A	
Do you monitor antibiotic use trends over time?	☐ Yes<br>☐ No<br>☐ N/A	
+Do you stratify data by unit?	☐ Yes<br>☐ No<br>☐ N/A	
+Do you stratify data by antibiotic/antibiotic class?	☐ Yes<br>☐ No<br>☐ N/A	
+Do you stratify data by clinician?	☐ Yes<br>☐ No<br>☐ N/A	
How are the data available to the ASP?	☐ Report provided at a predetermined interval by IT, pharmacy, etc.<br>What interval?<br>☐ Monthly<br>☐ Quarterly<br>☐ Annually<br>☐ Other:<br>☐ Data available in real-time on a dashboard<br>☐ Other:	
Does your ASP present antibiotic use data to the ASP Committee?	☐ Yes<br>☐ No<br>☐ N/A	

© 2021 Tamma D et al. *JAMA Network Open.*

ASP Area	Answers	Comments
**+Does your ASP present antibiotic use data to facility leadership?**	☐ Yes<br>☐ No<br>☐ N/A	
**+Does your ASP present antibiotic use data to frontline staff or unit directors?**	☐ Yes<br>☐ No<br>☐ N/A	
**+Do you report antibiotic use data to the National Healthcare Safety Network Antimicrobial Use and Resistance Module?**	☐ Yes<br>☐ No<br>☐ N/A	
**Other Metrics**		
**+Does the ASP measure the number and type of interventions performed?**	☐ Yes<br>☐ No	
**+Does the ASP identify units with high *Clostridioides difficile* rates and assess antimicrobial use on the units?**	☐ Yes<br>☐ No	
**Education**		
**Does your ASP provide updates to healthcare providers about judicious antibiotic prescribing and the role of antibiotic stewardship?**	☐ No<br>☐ Yes: Nurses<br>☐ Yes: Pharmacists<br>☐ Yes: Prescribers<br>☐ Yes: Other(s)	
**How frequently does your ASP provide updates to health care providers about judicious antibiotic prescribing and the role of antibiotic stewardship?**	☐ Annually<br>☐ Annually, and as needed<br>☐ Unscheduled<br>☐ Other:<br>☐ N/A	

AHRQ Pub. No. 17(20)-0028-EF
November 2019

© 2021 Tamma D et al. *JAMA Network Open.*

AHRQ Safety Program for Improving Antibiotic Use

Team Antibiotic Review Form

***Questions 1–6 should be answered for all patients you evaluate who are actively receiving antibiotics.**

Question 1: Day of antibiotic therapy (choose one)

☐Day 1 ☐Day 2 ☐Day 3 ☐Day 4 ☐Day 5 ☐Day 6 ☐> 7 Days

Question 2: Record antibiotic regimen and indication below:

Antibiotic: ____________________ Indication: ____________________
Antibiotic: ____________________ Indication: ____________________
Antibiotic: ____________________ Indication: ____________________
Antibiotic: ____________________ Indication: ____________________

Moment ONE			
**Question 3: Does the patient have a suspected or confirmed infection that requires antibiotics?**	☐ Yes	☐ No	

Moment TWO			
**Question 4: Were appropriate cultures ordered before antibiotics were started?**	☐ Yes	☐ No	
**Question 5: Were specific reactions for reported antibiotic allergies documented?**	☐ Yes	☐ No	☐ N/A
**Question 6: Were empiric antibiotics compliant with local guidelines?**	☐ Yes	☐ No	☐ N/A

© 2021 Tamma D et al. *JAMA Network Open.*

** For patients who have been receiving antibiotics longer than 24 hours, answer questions 7–14 in addition to the above questions 1–6.*

Moment THREE			
**Question 7: Are antibiotics still needed?**	☐ Yes	☐ No	
If you answered "no" to Question 7, answer Question 8. Otherwise go to Question 9.			
**Question 8: If antibiotics are not needed, will you stop them today?**	☐ Yes	☐ No	
**Question 9: Can antibiotics be narrowed based on microbiology data or other clinical data?**	☐ Yes	☐ No	☐ Already narrowed
If you answered "yes" to Question 9, answer Question 10. Otherwise go to Question 11.			
**Question 10: If antibiotics can be narrowed, will you change to a narrower regimen today?**	☐ Yes	☐ No	
**Question 11: Can antibiotics be changed from intravenous to oral?**	☐ Yes	☐ No	☐ Already on PO
If you answered "yes" to Question 11, answer Question 12. Otherwise go to Question 13.			
**Question 12: If antibiotics can be changed from intravenous to oral, will you change to oral therapy today?**	☐ Yes	☐ No	

Moment FOUR			
**Question 13: Has a planned duration been documented in the medical record?**	☐ Yes	☐ No	
If you answered "yes" to Question 13, answer Question 14. Otherwise this form has been completed.			
**Question 14: Is the planned duration consistent with local guidelines?**	☐ Yes	☐ No	☐ N/A

AHRQ Pub. No. 17(20)-0028-EF
November 2019

© 2021 Tamma, D. et al. *JAMA Network Open.*

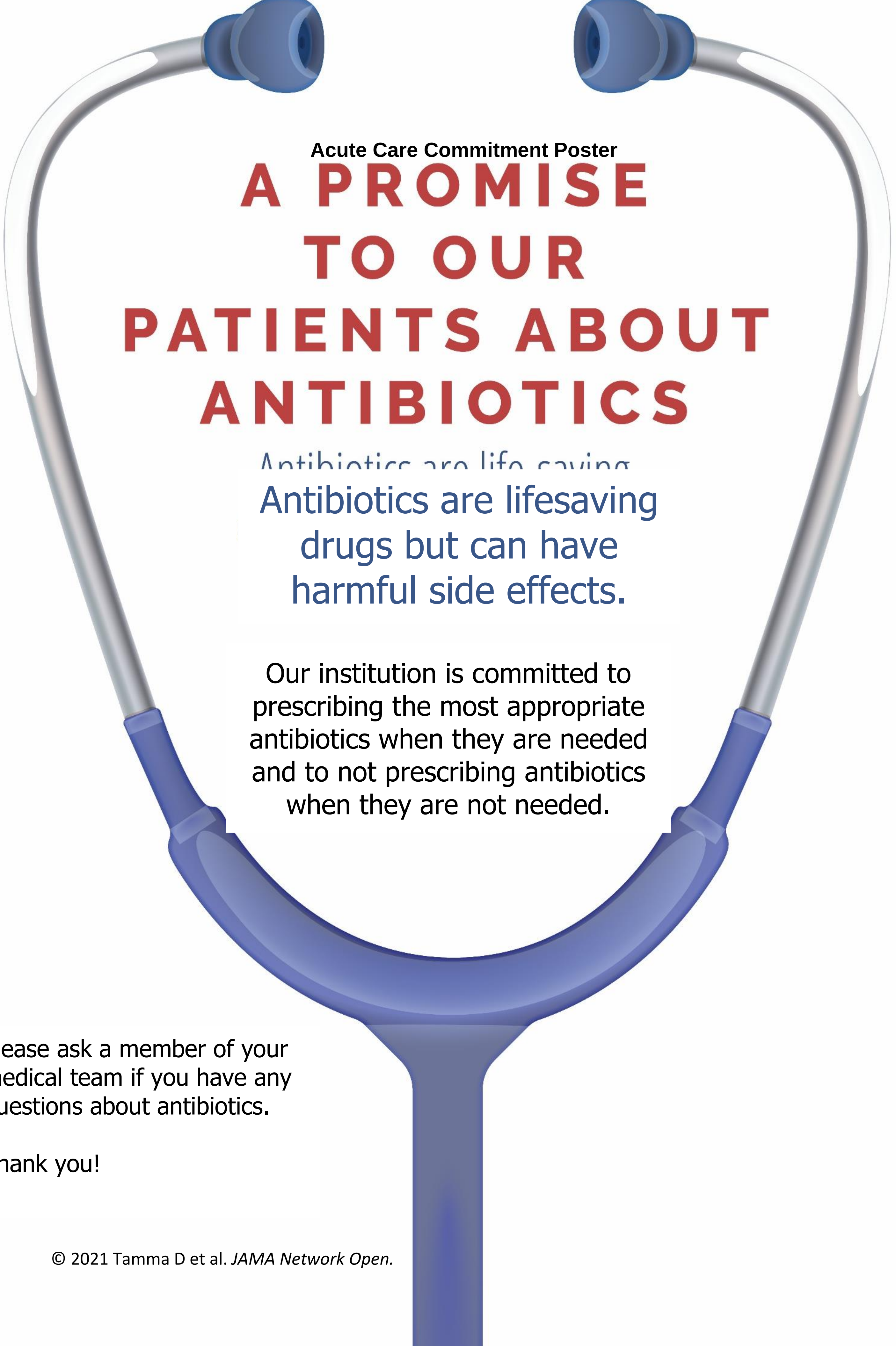

Acute Care Commitment Poster

A PROMISE TO OUR PATIENTS ABOUT ANTIBIOTICS

Antibiotics are life saving

Antibiotics are lifesaving drugs but can have harmful side effects.

Our institution is committed to prescribing the most appropriate antibiotics when they are needed and to not prescribing antibiotics when they are not needed.

Please ask a member of your medical team if you have any questions about antibiotics.

Thank you!

© 2021 Tamma D et al. *JAMA Network Open.*

Directions for Completing and Displaying the Acute Care Commitment Poster

How to "sign" the document

Once the document is open in Microsoft Word, click on the bottom right side of the document (the blank space) and you will find a text box (with no fill color or outline).

Use this text box to include the photographs and signatures of the individuals you believe are important to indicate your institution's commitment to prescribing antibiotics responsibly. If the poster is being displayed in a general area of the hospital, consider having executive leadership and other influential leaders (e.g., medical, pharmacy, and nursing administrators) sign it. If it is being displayed in a specific unit, consider having the unit executive and the medical, pharmacy, and nursing director of the unit sign it.

How to add a logo

If you choose to add a logo to this document, there are two placement options: in the signature textbox or on the footer (next to the AHRQ logo).

Add logo to signature box

1. Click in the text box (which has no fill color or outline) located at the bottom right space of the poster. Make sure your cursor is in the box.
2. Go to the "Insert" tab.
3. Click on "Pictures" from the "Insert" tab.
4. A window will pop up so you can search your computer for the logo. Select your logo (.jpg or .png format) and click the insert button.
5. Your logo will appear in the text box.

Add logo to footer (next to AHRQ logo)

1. Open the footer section (either double click in the footer section of the document or go to the "Insert" tab-> select the drop down menu for "Footer"-> and click on "Edit Footer").
2. Follow steps 2-4 from "Add logo to signature box."
3. Your logo will appear in your footer. If you want to move the logo image around, click on the logo-> go to the "Format" tab -> click on the "Wrap Text" dropdown menu -> and select "In front of text." This formatting will allow you to move the logo freely around the screen.
4. When you are satisfied with the placement of the logo, exit the footer section (either double click outside of the footer area or go to the "Design" tab-> and click the "Close header and footer" button).

© 2021 Tamma D et al. *JAMA Network Open*.

AHRQ Pub. No. 17(20)-0028-EF
November 2019

PREVENT HAIs Healthcare-Associated Infections

Antibiotic Time Out Tool

Date: ____________ Patient Name or Identifier: ______________________________

Directions: This form should be completed by frontline clinicians on a daily basis for patients receiving antibiotics.

Note: A table of commonly recommended durations of therapy is available on the back of this document.

Antibiotic 1: ______________________ Treatment day #: __________

Antibiotic 2: ______________________ Treatment day #: __________

Antibiotic 3: ______________________ Treatment day #: __________

Check the patient's indication(s) for continuing antibiotics below:

☐ Prophylaxis
☐ Central nervous system infection
☐ Head and neck infection
☐ Endovascular infection/endocarditis
☐ Community-acquired pneumonia
☐ Hospital-acquired pneumonia
☐ Ventilator-associated pneumonia
☐ *Clostridioides difficile* infection
☐ Biliary tract infection
☐ Diverticulitis
☐ Intra-abdominal infection
☐ Urinary tract infection (UTI)
☐ Osteoarticular infection
☐ Skin/soft tissue infection
☐ Sepsis, unknown source
☐ Bacteremia
☐ Other:

Is the patient receiving antibiotics for any of the following conditions even though antibiotics are NOT typically recommended?

☐ Positive urine culture without symptoms of a UTI (Exceptions: pregnancy or impending urologic surgery where mucosal bleeding is expected)
☐ *Enterococcus* in sputum
☐ Coagulase-negative staphylococci in a single blood culture
☐ *Candida* in sputum or urine
☐ Surgical prophylaxis beyond 24 hours
☐ Noninfectious etiology of symptoms

Answer Yes or No questions below based on patient's clinical status and culture results.

Can any of the antibiotics be discontinued?	☐ Yes	☐ No
Can existing therapy be changed to a more narrow spectrum regimen?	☐ Yes	☐ No
Should additional agents or broader-spectrum agents be added?	☐ Yes	☐ No
Are there any IV agents that can be changed to the PO route?	☐ Yes	☐ No
Are the antibiotics selected consistent with local guidelines?	☐ Yes	☐ No

What is the planned duration of antibiotic therapy?

Antibiotic 1: ____________________ Planned duration: _________ Consistent with recommended duration? ☐Yes ☐No

Antibiotic 2: ____________________ Planned duration: _________ Consistent with recommended duration? ☐Yes ☐No

Antibiotic 3: ____________________ Planned duration: _________ Consistent with recommended duration? ☐Yes ☒No

© 2021 Tamma D et al. *JAMA Network Open*.

AHRQ Agency for Healthcare Research and Quality

Infectious process	Specific agents/circumstances	Recommended duration of antibiotic therapy*
**Community-acquired pneumonia**	**n/a**	5 days[1-3]
**Hospital-acquired or healthcare-associated pneumonia**	**n/a**	7 days[4,5]
**Ventilator-associated pneumonia**	**n/a**	7 days[4,5]
**Cystitis**	Nitrofurantoin or cephalosporin	5 days[6-8]
	Trimethoprim/sulfamethoxazole (TMP/SMX)	3 days[6-9]
**Pyelonephritis**	Fluoroquinolone	5–7 days[6,10-12]
	TMP/SMX or oral cephalosporin	10-14 days[6,11] (shorter course if early response)
**Complicated urinary tract infection (UTI), including catheter-associated UTI (CAUTI)**	Lower tract CAUTI in women ≤ 65 years if catheter is removed	3 days[13,14]
	Prompt resolution of symptoms	7 days[14]
	Delayed response, obstruction or other urologic abnormality	10–14 days[14]
**Skin and soft-tissue infection**	Clinical response by day 3	5–7 days[15]
**Diverticulitis**	Acute, uncomplicated	0–4 days[16,17]
	Complicated or initial severe illness with source control	4 days after source control[18]
	Complicated with small abscess, not drained*	5–10 days based on clinical response[15,19]
**Biliary tract infection**	Acute cholangitis and source control	3 days after source control[20,21]
	Acute cholangitis and source control with concomitant bacteremia	7 days[22]
	Uncomplicated acute cholecystitis, medical management*	5–10 days based on clinical response[15,19]
	Uncomplicated acute cholecystitis, surgical management	No antibiotics after surgery[23]
	Complicated acute cholecystitis (e.g., perforation, fistula), surgical management for source control	4 days after surgery[18]
**Intra-abdominal infection with source control**	**n/a**	4 days[18]
**Gram-negative bloodstream infection with source control**	**n/a**	7 days[24]

*For all durations, recommendations are for patients without significant immunocompromise or complex presentations; relevant multi-specialty consultation, including infectious diseases, should be considered for cases falling outside of the scope of these recommendations.

© 2021 Tamma D et al. *JAMA Network Open.*

References

1. Metlay JP, Waterer GW, Long AC, et al. Diagnosis and treatment of adults with community-acquired pneumonia. An official clinical practice guideline of the American Thoracic Society and Infectious Diseases Society of America. Am J Respir Crit Care Med. 2019 Oct 1;200(7):e45-e67. PMID: 31573350.
2. el Moussaoui R, de Borgie CA, van den Broek P, et al. Effectiveness of discontinuing antibiotic treatment after three days versus eight days in mild to moderate-severe community acquired pneumonia: randomized, double blind study. BMJ. 2006 Jun 10;332(7554):1355. PMID: 16763427.
3. Uranga A, España PP, Bilbao A, et al. Duration of antibiotic treatment in community-acquired pneumonia: a multicenter randomized clinical trial. JAMA Intern Med. 2016 Sep 1;176(9):1257-65. PMID: 27455166.
4. Chastre J, Wolff M, Fagon JY, et al. Comparison of 8 vs 15 days of antibiotic therapy for ventilator-associated pneumonia in adults: a randomized trial. JAMA. 2003 Nov 19;290(19):2588-98. PMID: 14625336.
5. Kalil AC, Metersky ML, Klompas M, et al. Management of adults with hospital-acquired and ventilator-associated pneumonia: 2016 Clinical Practice Guidelines by the Infectious Diseases Society of America and the American Thoracic Society. Clin Infect Dis. 2016 Sep1;63(5)e61-111. PMID: 27418577.
6. Gupta K, Hooton TM, Naber KG, et al. International clinical practice guidelines for the treatment of acute uncomplicated cystitis and pyelonephritis in women: a 2010 update by the Infectious Diseases Society of America and the European Society for Microbiology and Infectious Diseases. Clin Infect Dis. 2011 Mar 1;52(5):e103-20. PMID: 21292654.
7. Gupta K, Hooton TM, Roberts PL, et al. Short-course nitrofurantoin for the treatment of acute uncomplicated cystitis in women. Arch Intern Med. 2007 Nov 12;167:2207-12. PMID: 17998493.
8. Huttner A, Verhaegh EM, Harbath S, et al. Nitrofurantoin revisited: a systematic review and meta-analysis of controlled trials. J Antimicrob Chemother. 2015 Sep;70(9):2456-64. PMID: 26066581.
9. Kavatha D, Giamarellou H, Alexiou Z, et al. Cefpodoxime-proxetil versus trimethoprim-sulfamethoxazole for short-term therapy of uncomplicated acute cystitis in women. Antimicrob Agents Chemother. 2003 Mar;47(3):897-900. PMID: 12604518.
10. Peterson J, Kaul S, Khashab M, et al. A double-blind, randomized comparison of levofloxacin 750 mg once-daily for five days with ciprofloxacin 400/500 mg twice-daily for 10 days for the treatment of complicated urinary tract infections and acute pyelonephritis. Urology. 2008 Jan;71(1):17-22. PMID: 18242357.
11. Talan DA, Stamm WE, Hooton TM, et al. Comparison of ciprofloxacin (7 days) and trimethoprim-sulfamethoxazole (14 days) for acute uncomplicated pyelonephritis in women: a randomized trial. JAMA. 2000 Mar 22-29;283(12):1583-90. PMID: 10735395.
12. Talan DA, Klimberg IW, Nicolle LE, et al. Once daily, extended release ciprofloxacin for complicated urinary tract infections and acute uncomplicated pyelonephritis. J. Urol. 2004 Feb;171(2 pt 1):734-9. PMID: 14713790.
13. Harding GK, Nicolle LE, Ronald AR, et al. How long should catheter-acquired urinary tract infection in women be treated? A randomized controlled study. Ann Intern Med. 1991 May 1;114(9):713-9. PMID: 2012351.
14. Hooton TM, Bradley SF, Cardenas DD, et al. Diagnosis, prevention, and treatment of catheter associated urinary tract infection in adults: 2009 international clinical practice guidelines from the Infectious Diseases Society of America. Clin Infect Dis. 2010 Mar;50(5):625-63. PMID: 20175247.
15. Stevens DL, Bisno AL, Chambers HF, et al. Practice guidelines for the diagnosis and management of skin and soft tissue infections: 2014 update by the Infectious Diseases Society of America. Clin Infect Dis. 2014 Jul 15:59(2)e10-52. PMID: 24973422.
16. Stollman N, Smalley W, Hirano I, et al. American Gastroenterological Association guideline on the management of acute diverticulitis. Gastroenterology. 2015 Dec;149(7):1944-9. PMID: 26453777.
17. Schug-Pass C, Geers P, Hügel O, et al. Prospective randomized trial comparing short-term antibiotic therapy versus standard therapy for acute uncomplicated sigmoid diverticulitis. Int J Colorectal Dis. 2010 Jun;25(6):751-9. PMID: 20140619.
18. Sawyer RG, Claridge JA, Nathens AB, et al. Trial of short-course antimicrobial therapy for intraabdominal infection. N Engl J Med. 2015 May 21;372(21):1996-2005. PMID: 25992746.
19. Mazuki JE, Tessier JM, May KM, et al. The Surgical Infection Society revised guidelines on the management of intra-abdominal infection. Surg Infect (Larchmt). 2017 Jan;18(1):1-76. PMID: 28085573.
20. van Lent AU, Bartelsman JF, Tytgat GN, et al. Duration of antibiotic therapy for cholangitis after successful endoscopic drainage of the biliary tract. Gastrointest Endosc. 2002 Apr;55(4):518-22. PMID: 11923764.
21. Kogure H, Tsujino T, Yamamoto K, et al. Fever-based antibiotic therapy for acute cholangitis following successful endoscopic biliary drainage. J Gastroenterol. 2011 Dec;46(12):1411-7. PMID: 21842232.
22. Chotiprasiakul D, Han JH, Cosgrove SE, et al. Comparing the outcomes of adults with *Enterobacteriaceae* bacteremia

© 2021 Tamma D et al. *JAMA Network Open.*

receiving short-course versus prolonged-course antibiotic therapy in a multicenter, propensity score-matched cohort. Clin Infect Dis. 2018 Jan 6:66(2):172-7. PMID: 29190320.

23. Regimbeau JM, Fuks D, Pautrat K, et al. Effect of postoperative antibiotic administration of postoperative infection following cholecystectomy for acute calculous cholecystitis: a randomized clinical trial. JAMA. 2014 Jul;312(2):145-54. PMID: 25005651.

24. Yahav D, Franceschini E, Koppel F, et al. Seven versus fourteen days of antibiotic therapy for uncomplicated Gram-negative bacteremia: a non-inferiority randomized controlled trial. Clin Infect Dis. 2019 Sep 13;69(7):1091-1098. PMID: 30535100.

AHRQ Pub. No. 17(20)-0028-EF
November 2019

© 2021 Tamma D et al. *JAMA Network Open.*

Antibiotic Use Submission Template

Monthly DOT

General Instructions:

- We are requesting two spreadsheets per quarter for each unit participating in the AHRQ Safety Program for Improving Antibiotic Use, one is monthly antibiotic use data, the other is quarterly CDI event data. Separate spreadsheets will be available for each quarter. This spreadsheet is for data collected during Quarter 1 (1/1/2018-3/31/2018).
- Please make sure the hospital and unit names are consistent across the two sheets (Monthly DOT, Quarterly C. difficile) and different cycles of submissions.
- The predefined cells of this worksheet are locked and protected (Column A, B4--D6). Please avoid editing these cells or changing their orders. If no patient is administered for an antibiotics across all three months, please leave the cells empty or fill in zeros.
- Before uploading this to the website, save it as "DOT Q1_Hospital Name_Unit Name." Insert your own hospital and unit name.

Instructions for Reporting Antibiotic Data:

- Total number of patient-days (B7, C7, D7) are the monthly totals of number of patients present in the unit at the same time (e.g. at midnight) of each day, summed across all days in the month. Please see Appendix 1 for examples.
- B8-B57, C8-C57, and D8-D57 reflect the aggregate number of days patients were administered each of the antibiotics in Column A within the time frame specified in B5-B6, C5-C6, and D5-D6 respectively. For example, if your unit had 3 patients using Amikacin in January 2018 and they used it for 3, 5, & 7 days, respectively, then days of Amikacin in January 2018 should be counted as 3+5+7=15 days and you should fill 15 in cell B8. If a patient used an antibiotic across months - for example, from 1/31/2017 to 2/2/2018 - then 1 day should be counted to January 2018 and 2 days should be counted towards February 2018.
- Please see NDC codes for corresponding antibiotics in Appendix 2.
- If you anticipate difficulties in collecting antibiotic usage data for the antibiotics listed below, please contact your Implementation Advisor for assistance.

**Hospital name**	**[insert hospital name]**		
**Unit name**	**[insert unit name]**		
**Month**	**January**	**February**	**March**
**Time starts**	1/1/2018	2/1/2018	3/1/2018
**Time ends**	1/31/2018	2/28/2018	3/31/2018
**Total number of patient-days each month**	[insert total # of patient-days for **January** here]	[insert total # of patient-days for **February** here]	[insert total # of patient-days for **March** here]
AMIKACIN	[insert aggregate # of days patients were administered Amikacin in participating unit in January here]	[insert aggregate # of days patients were administered Amikacin in participating unit in February here]	[insert aggregate # of days patients were administered Amikacin in participating unit in March here]
AMOXICILLIN			

© 2021 Tamma D et al. *JAMA Network Open.*

AMOXICILLIN/CLAVULANATE			
AMPICILLIN			
AMPICILLIN/SULBACTAM			
AZITHROMYCIN			
AZTREONAM			
CEFACLOR			
CEFAZOLIN			
CEFEPIME			
CEFOTAXIME			
CEFOTETAN			
CEFOXITIN			
CEFTAROLINE			
CEFTAZIDIME			
CEFTAZIDIME/AVIBACTAM			
CEFTOLOZANE/TAZOBACTAM			
CEFTRIAXONE			
CEFUROXIME			
CIPROFLOXACIN			
CLARITHROMYCIN			
CLINDAMYCIN			
COLISTIMETHATE			
DAPTOMYCIN			
DORIPENEM			
DOXYCYCLINE			
ERTAPENEM			
FIDAXOMICIN			
FOSFOMYCIN			
GENTAMICIN			
IMIPENEM/CILASTATIN			
LEVOFLOXACIN			
LINEZOLID			
MEROPENEM			
MEROPENEM/VABORBACTAM			
METRONIDAZOLE			
MOXIFLOXACIN			
NAFCILLIN			
NITROFURANTOIN			
OXACILLIN			
PENICILLIN G			

© 2021 Tamma D et al. *JAMA Network Open.*

PIPERACILLIN/TAZOBACTAM			
POLYMYXIN B			
RIFAMPIN			
SULFAMETHOXAZOLE/TRIMETHOPRIM			
TEDIZOLID			
TELAVANCIN			
TIGECYCLINE			
TOBRAMYCIN			
VANCOMYCIN			

Calculate Pt-Days

This example illustrates two methods to calculate the number of patient days

The example shows a case of 5 patients and set the time frame as 1/1/2018-1/5/2018

Both methods calculate the total number of patient days as 18 days among the 5 patients during 1/1/2018-1/5/2018

Patient	Admitted or transferred in to the unit	Discharge or transferred out from the unit
A	1/1/18 12:00 AM	1/5/18 2:00 PM
B	1/1/18 4:00 PM	1/4/18 12:01 AM
C	1/1/18 8:00 PM	1/7/18 11:59 PM
D	1/2/18 3:00 AM	1/6/18 5:00 AM
E	1/2/18 6:00 AM	1/8/18 6:00 AM

Method 1: Count number of patients in the unit at the same time for each day in the given time frame and sum across all days

Time	# of patients in the unit
1/1/18 12:00 AM	1 (patient A)
1/2/18 12:00 AM	3 (patient A, B, C)
1/3/18 12:00 AM	5 (patient A, B, C, D, E)
1/4/18 12:00 AM	5 (patient A, B, C, D, E)
1/5/18 12:00 AM	4 (patient A, C, D, E)

Method 2: Count days contributed by each patient in a given time frame and sum across all patients

Patient	Patient-days between 1/1/2018 and 1/5/2018
A	5 days (The patient is in the unit at 12:00 AM on 1/1, 1/2, 1/3, 1/4, and 1/5)
B	3 days (The patient is in the unit at 12:00 AM on 1/2, 1/3, and 1/4)
C	4 days (The patient is in the unit at 12:00 AM on 1/2, 1/3, 1/4, and 1/5)
D	3 days (The patient is in the unit at 12:00 AM on 1/3, 1,4 and 1/5)
E	3 days (The patient is in the unit at 12:00 AM on 1/3, 1,4 and 1/5)

© 2021 Tamma D et al. *JAMA Network Open.*

Quarterly Benchmarking Report, 4th Quarter, October - December 2018

Facility: ABC Medical Center
Unit: Medical Floor
Hospital Benchmark: all participating units from Community Hospitals with less than 300 beds
Unit Benchmark: all participating Medical units/wards from all participating hospitals

As part of participation in the AHRQ Safety Program for Improving Antibiotic Use, your unit will receive quarterly benchmarking reports to compare your unit's progress to those of units in similar facilities.

This report contains individualized results from all the data submitted by your unit for the 1st, 2nd, 3rd, and 4th quarters (January – December 2018). It includes the following results for your unit:

•	1st quarter antibiotic days of therapy (DOT)
•	2nd quarter antibiotic days of therapy (DOT)
•	3rd quarter antibiotic days of therapy (DOT)
•	4th quarter antibiotic days of therapy (DOT)
•	1st quarter *C. difficile* LabID events
•	2nd quarter *C. difficile* LabID events
•	3rd quarter *C. difficile* LabID events
•	4th quarter *C. difficile* LabID events
•	March - May 2018 Team Antibiotic Review Forms
•	June - August 2018 Team Antibiotic Review Forms
•	September - November 2018 Team Antibiotic Review Forms

The report also includes aggregate data results from all participating units in similar facilities (Hospital Benchmark) and from similar units in all participating hospitals (Unit Benchmark). Both benchmarks include data available at the time of production of this report. The benchmarks and your unit's relation to these benchmarks may have changed from previous quarterly reports as more data from participating facilities has been included.

If your unit submitted data that are not specific to your registered unit, incomplete quarterly data (e.g., missing data for one month), a denominator other than patient-days (e.g., days-present), and/or out-of-range data (low or high patient-days/rates in comparison to the benchmark), your unit's data will be excluded from the benchmark calculation as they are not directly comparable to benchmark data. Please see individual results below for more detail.

© 2021 Tamma D et al. *JAMA Network Open.*

Please note that results from individual units will not be shared with other participating hospitals; the report only includes aggregate benchmark data from other hospitals. We welcome your feedback on the report. If you have any questions about the report, or the individual results from your unit, please contact your implementation adviser.

© 2021 Tamma D et al. *JAMA Network Open.*

C. difficile LabID Events

C. difficile LabID events per 10,000 patient-days
The following figure shows the trend of number of *C. difficile* LabID events per 10,000 patient-days in your unit, all participating units from Community Hospitals with less than 300 beds, and Medical units/wards from all participating hospitals. The benchmark rates represent average rates across all included units.

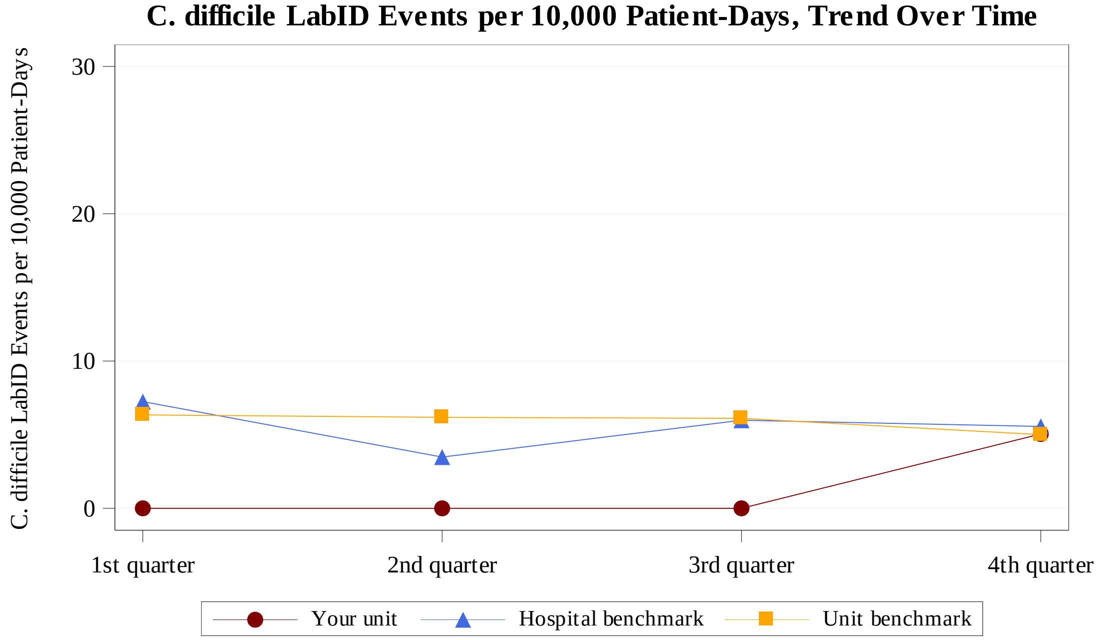

	1st quarter	2nd quarter	3rd quarter	4th quarter
**Your unit**	0	0	0	5
**Hospital benchmark**	7.2	3.5	6	5.6
**Unit benchmark**	6.3	6.2	6.1	5

The number of units submitting Q4 data for your unit's benchmarking cohorts are as follows:

- Hospital Benchmark: 82 units from 69 hospitals
- Unit Benchmark: 64 units from 59 hospitals

The following results compare your unit's rate to all rates in your unit's benchmark cohorts:

- Compared to individual units in your hospital benchmark, your unit's rate of *C. difficile* LabID events is higher than at least 50% of similar units.
- Compared to individual units in your unit benchmark, your unit's rate of *C. difficile* LabID events is higher than at least 50% of similar units.

© 2021 Tamma D et al. *JAMA Network Open.*

Antibiotic Use Data

Antibiotic days of therapy (DOT) per 1,000 patient-days

The following figure shows the trend of monthly days of therapy per 1,000 patient-days in your unit, all participating units from Community Hospitals with less than 300 beds, and Medical units/wards from all participating hospitals. It includes data for all antibiotics reported by your unit. The benchmark rates represent an average rate across all included units.

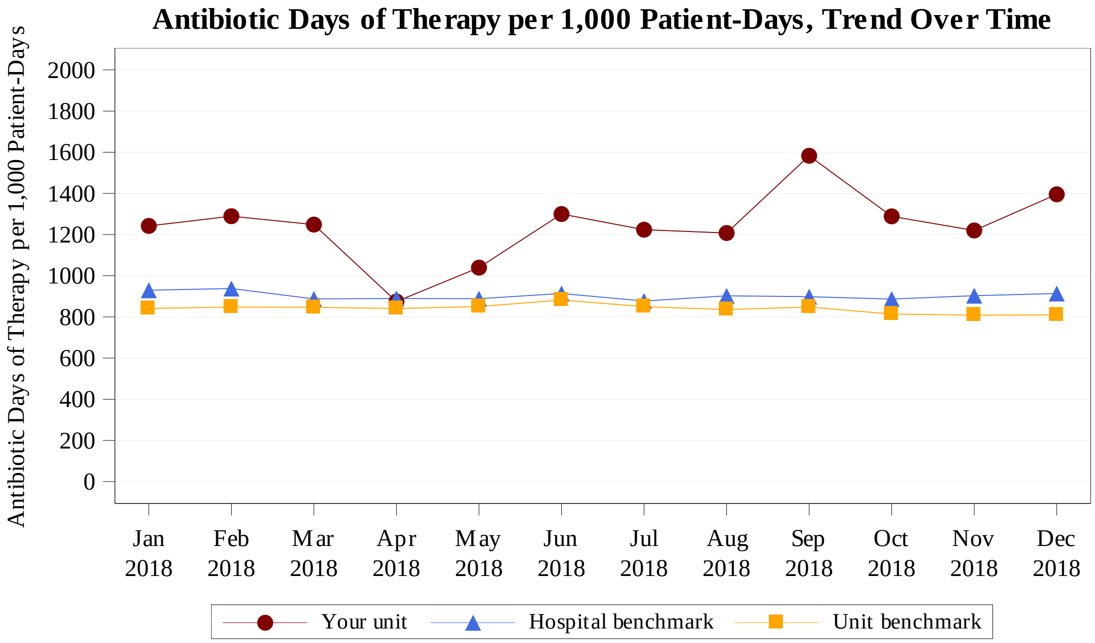

**Your unit**	1242	1289	1249	875	1039	1300	1224	1208	1583	1289	1220	1396
**Hospital benchmark**	930	938	888	890	889	914	877	903	898	887	903	914
**Unit benchmark**	841	848	847	841	850	882	850	836	848	814	809	810

The number of units submitting Q4 data for your unit’s benchmarking cohorts are as follows:

- Hospital Benchmark: 87 units from 74 hospitals
- Unit Benchmark: 67 units from 62 hospitals

The following results compare your unit's rate to all rates in your unit's benchmark cohort:

- Compared to individual units in your hospital benchmark, your unit's rate of days of therapy is higher than at least 75% of similar units.
- Compared to individual units in your unit benchmark, your unit's rate of days of therapy is higher than at least 75% of similar units.

2021 Tamma D et al. *JAMA Network Open.*

The figure below shows the quarterly antibiotic days of therapy per 1,000 patient-days from Q1 to each subsequent program quarter, for your unit and for the hospital and unit benchmarks, respectively. The benchmark rates represent an average rate across all included units.

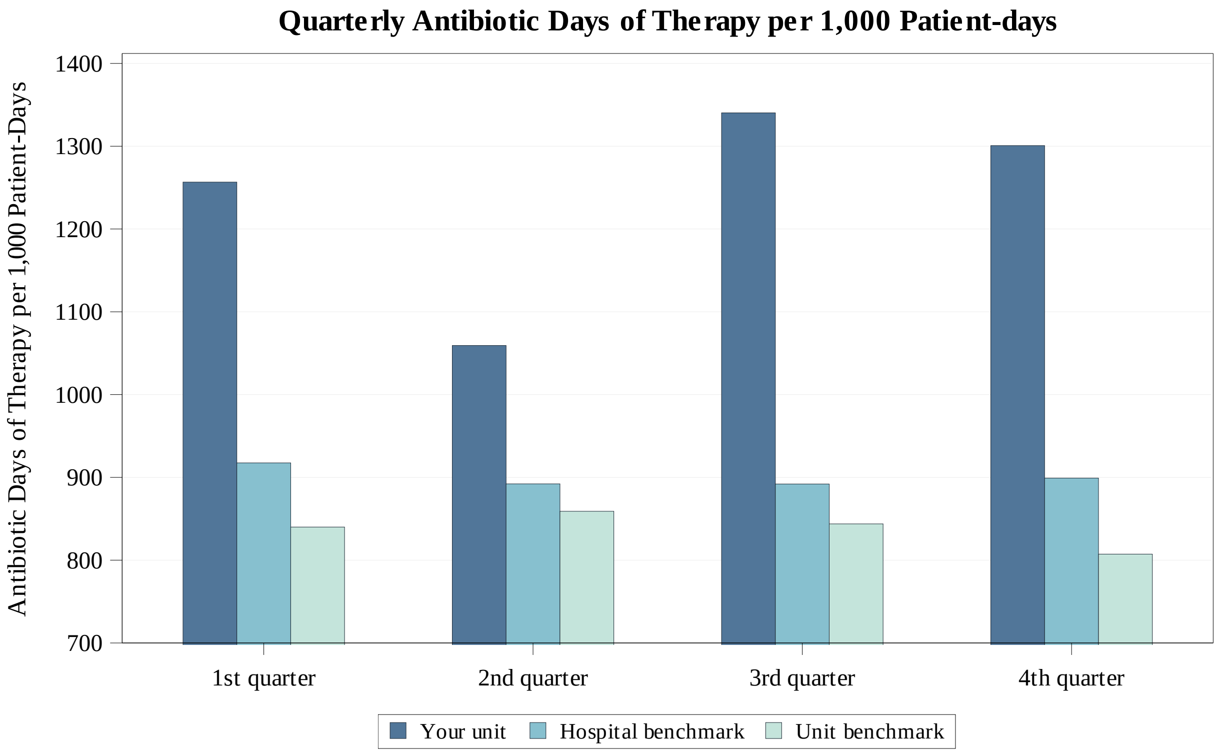

© 2021 Tamma D et al. *JAMA Network Open.*

The following figures show the quarterly days of therapy per 1,000 patient-days for antibiotics in each of five drug classes of interest. Data are shown for the hospital benchmark, unit benchmark, and your unit.

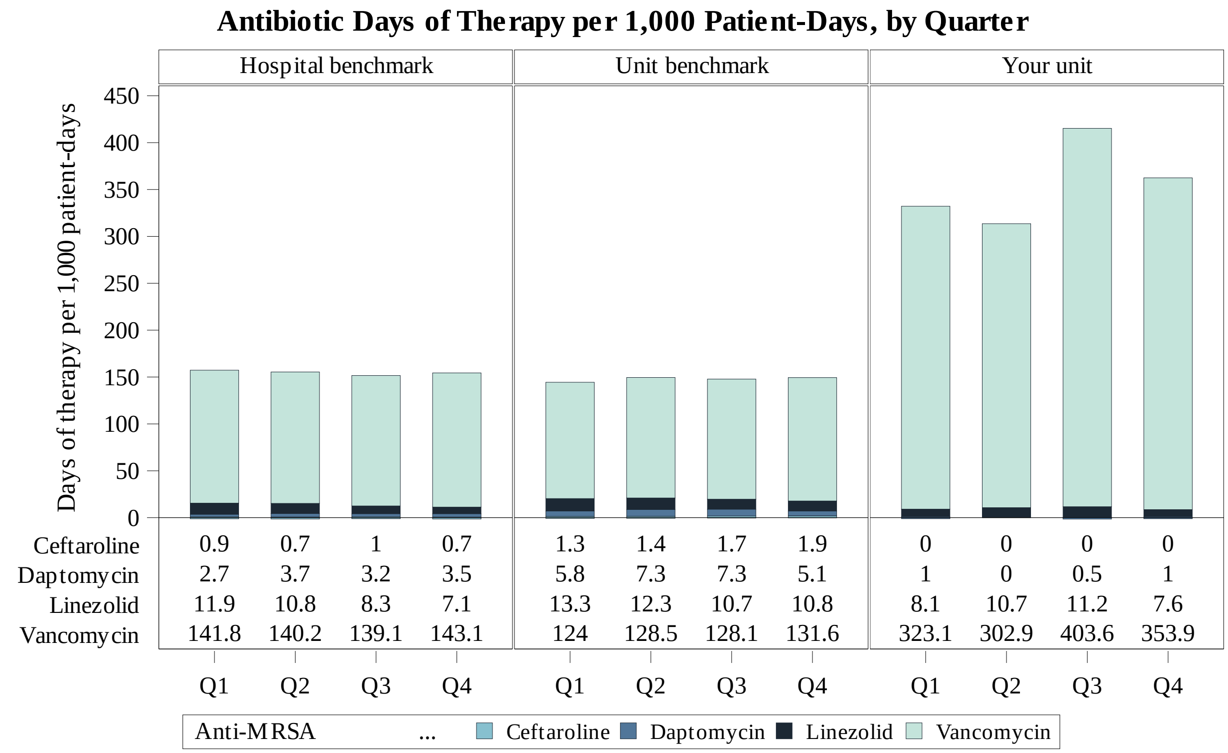

	Hospital benchmark Q1	Q2	Q3	Q4	Unit benchmark Q1	Q2	Q3	Q4	Your unit Q1	Q2	Q3	Q4
Ceftaroline	0.9	0.7	1	0.7	1.3	1.4	1.7	1.9	0	0	0	0
Daptomycin	2.7	3.7	3.2	3.5	5.8	7.3	7.3	5.1	1	0	0.5	1
Linezolid	11.9	10.8	8.3	7.1	13.3	12.3	10.7	10.8	8.1	10.7	11.2	7.6
Vancomycin	141.8	140.2	139.1	143.1	124	128.5	128.1	131.6	323.1	302.9	403.6	353.9

© 2021 Tamma D et al. *JAMA Network Open.*

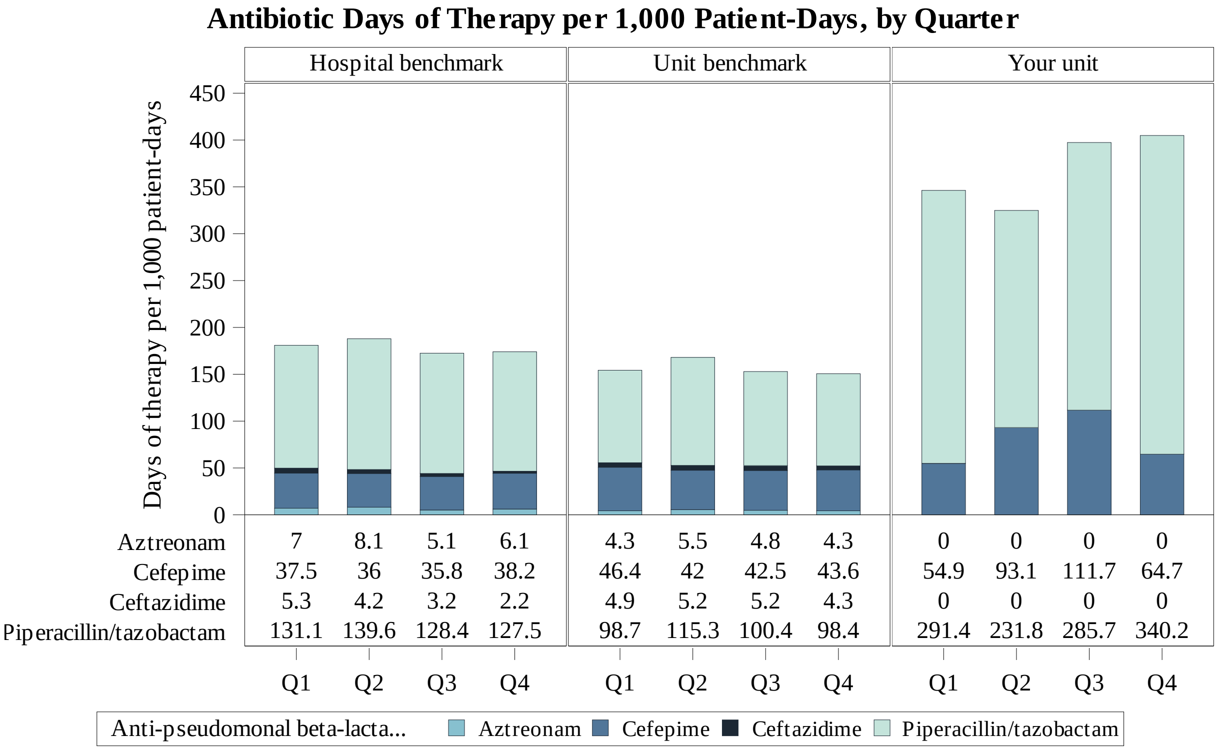

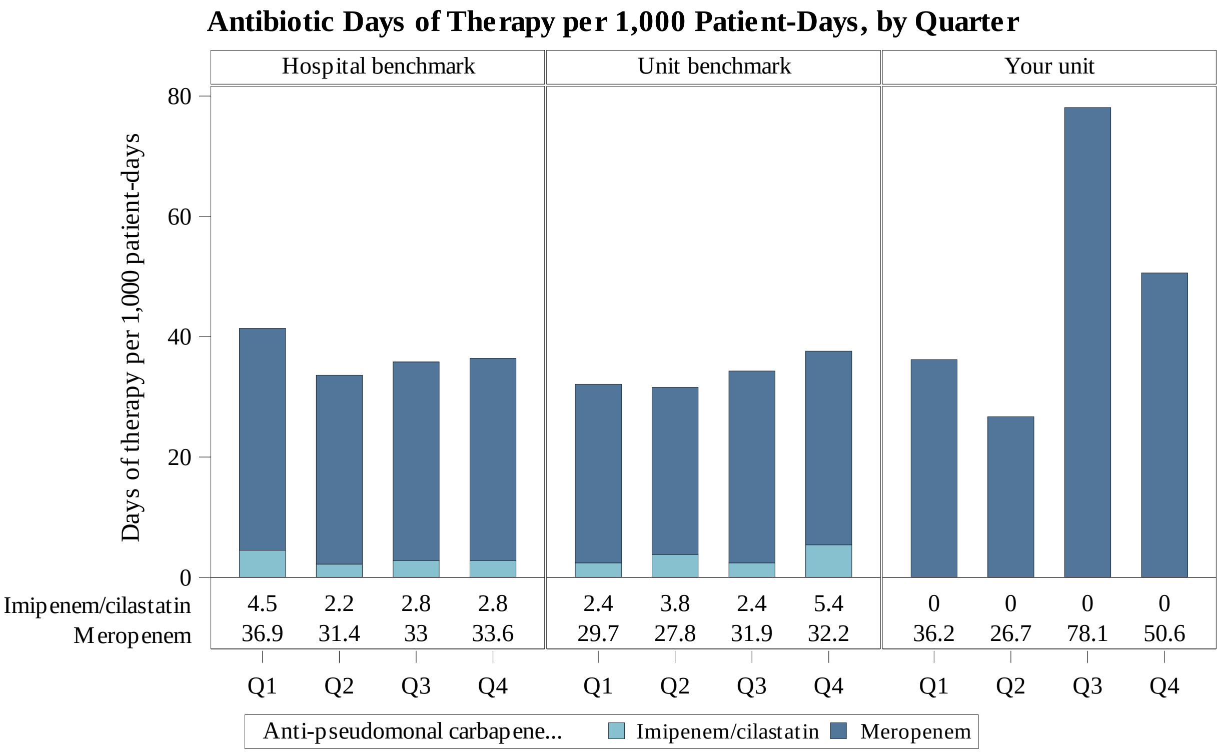

© 2021 Tamma D et al. *JAMA Network Open.*

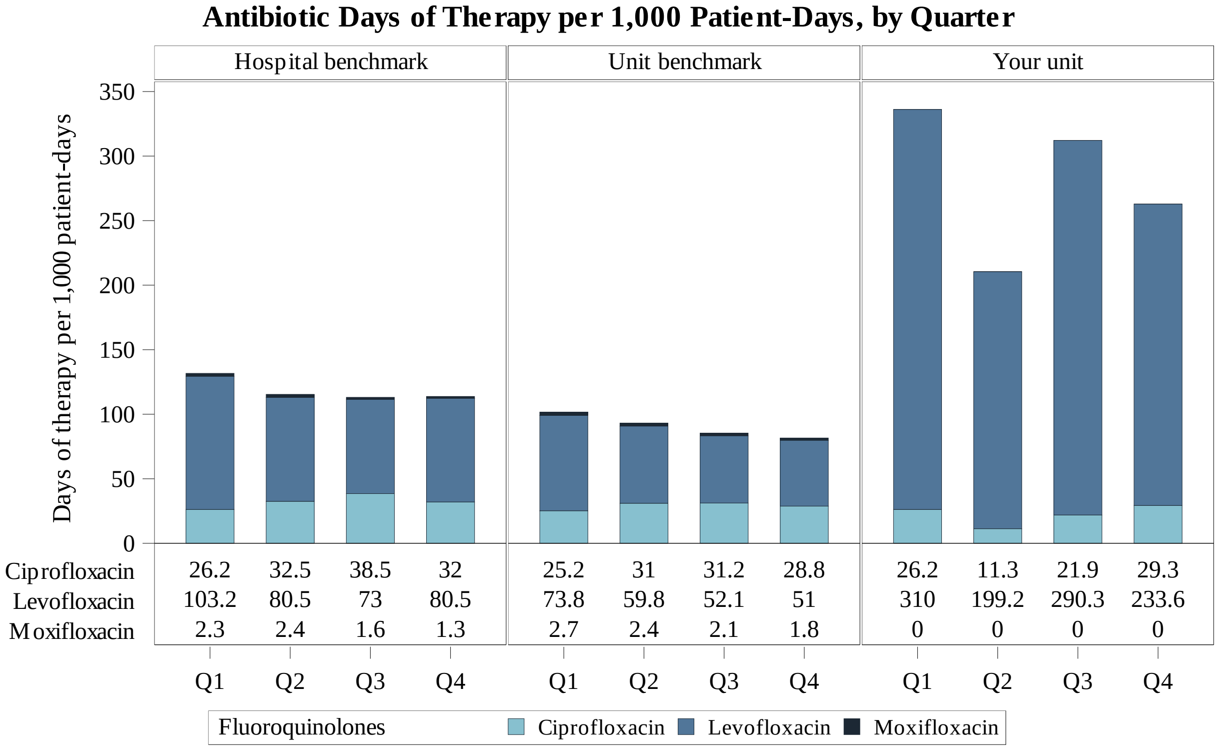

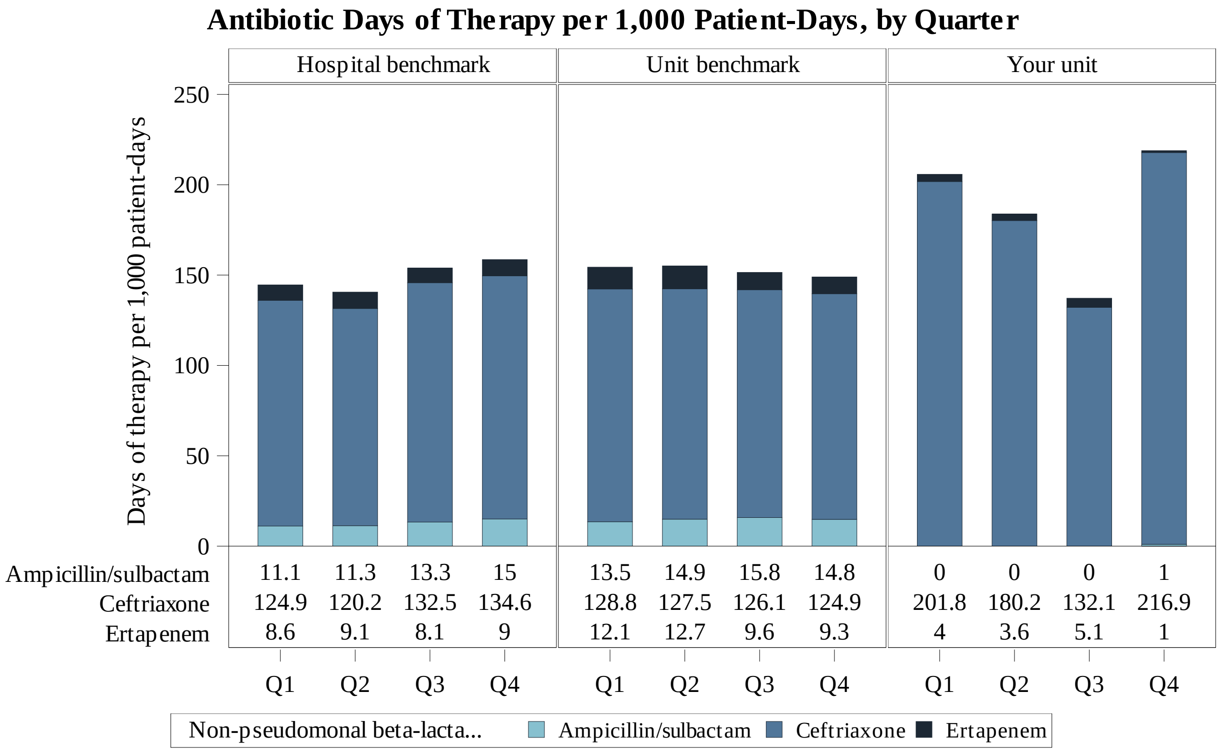

© 2021 Tamma D et al. *JAMA Network Open.*

Team Antibiotic Review Form

See program website for team antibiotic use review form

Your unit submitted Team Antibiotic Review Forms for 9 months, with an average of 10 antibiotic use review forms per month from March to November 2018. The graph below shows the summary of responses reported on the Team Antibiotic Review Forms, by quarter, for your unit. Each bar represents the percentage of submitted forms answering "yes" to a particular question or set of questions related to the patients on antibiotics that were evaluated. Responses of "not applicable" are not included in the graph.

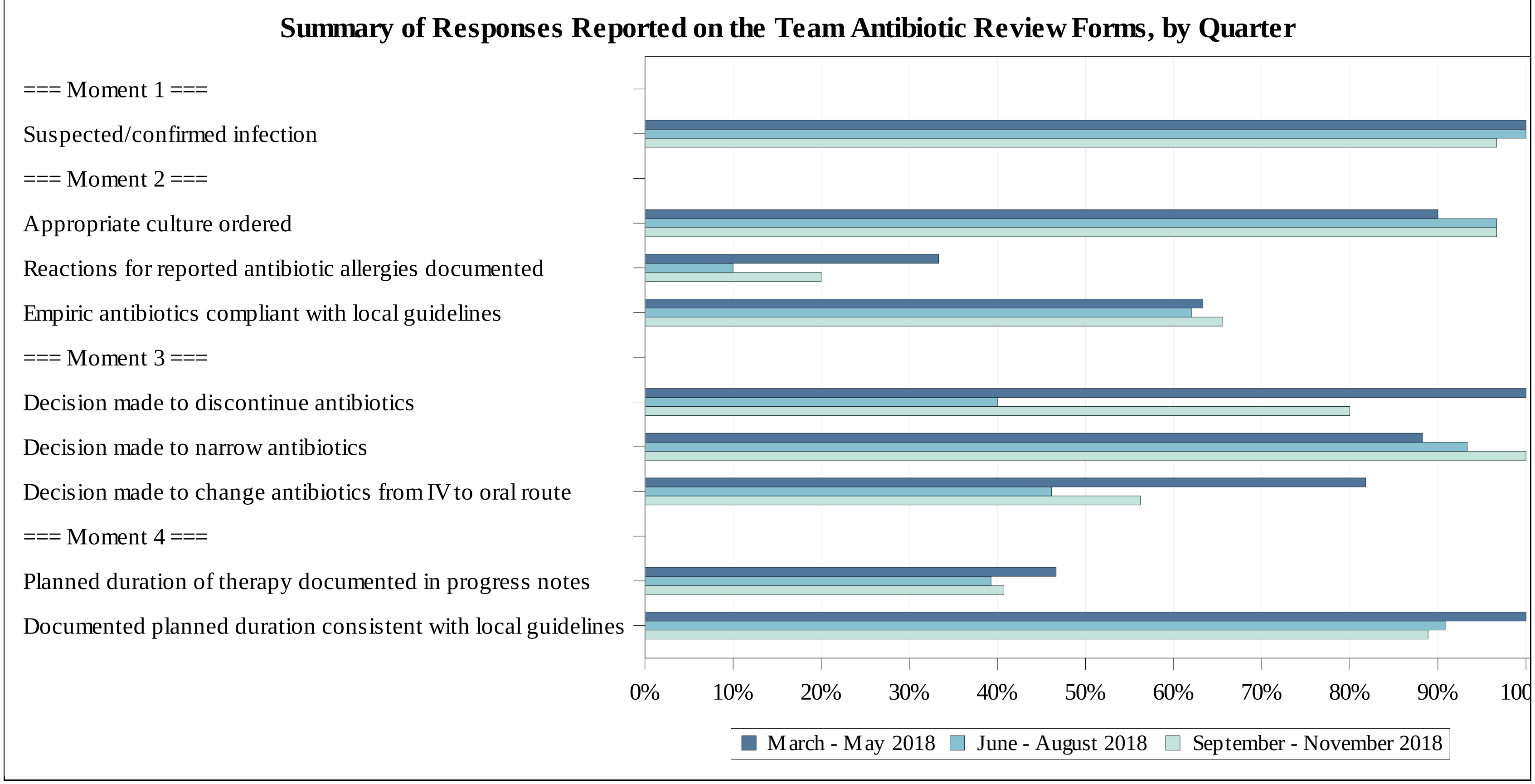

© 2021 Tamma D et al. *JAMA Network Open.*

PREVENT HAIs Healthcare-Associated Infections

Toolkit Implementation Guide for Acute Care Antibiotic Stewardship Programs

Introduction

Developing an antibiotic stewardship program (ASP) or improving an existing ASP can take time. If you are starting a program or growing a nascent program, the resources provided in the AHRQ Safety Program toolkit are intended to be introduced and implemented over several months. If you have an existing ASP, you should assess what elements of the toolkit will improve your program. Regardless of the stage of your ASP, you should begin by reviewing all elements of the toolkit, described below.

Develop and Improve Your Stewardship Program

It may be most useful to begin with the first four presentations under the "Develop and Improve your Stewardship Program" tab to ensure that you have the basics in place for a functioning ASP. Each presentation throughout the toolkit includes both a slide set and a script, referred to in the toolkit as a facilitator guide. The presentations in this section are directed at ASP leaders and cover developing an ASP, determining core interventions that the ASP will perform on a regular basis, measuring the success of the ASP, managing behavior change as a steward, and sustaining an ASP.

The two presentations on developing an ASP (part 1 and part 2) and the gap analysis tool can be used to determine what areas of your program may benefit from improvement. If after completing the gap analysis, you note major deficiencies in your ASP, particularly those that might lead to noncompliance with The Joint Commission Antimicrobial Stewardship Standard or other similar standards, you should meet with hospital leadership to determine how to manage the deficiencies. This may include developing a business case for additional physician or pharmacist resources or gaining access to data analysis resources.

All stewards should view the presentation "Making Effective Behavior Changes Around Antibiotic Prescribing," which provides an overview of behavioral aspects of antibiotic stewardship and practical approaches to modify prescriber behavior.

Once you have completed this work, consider viewing the two narrated presentations in this section regarding collaboration with bedside nurses and the microbiology lab for ideas about how to integrate the work of these important stakeholders into ASP practice.
© 2021 Tamma D et al. *JAMA Network Open*.

Four Moments of Antibiotic Decision Making Framework

Next, review the Four Moments of Antibiotic Decision Making framework and determine how to present it to frontline clinicians at your hospital. This framework identifies the critical time periods of antibiotic decision making throughout a course of antibiotics. It is intended to be disseminated to all frontline clinicians to ensure a rational thought process is employed when making decisions about whether antibiotics are needed and if so, what the most appropriate regimen is. Even if you have a robust ASP, consider taking the additional step of introducing the Four Moments framework so that frontline clinicians can be active participants in the process of improving antibiotic prescribing.

Several actions can be taken to integrate the Four Moments into regular practice:

- Local guidelines should be developed using the Four Moments framework. Thus, guidelines should use appropriate diagnostic criteria to determine if a patient has an infection, common causative organisms and cultures that should be obtained, recommendations for empiric therapy, recommendations for narrowing therapy and transitioning from intravenous to oral therapy, and recommendations for duration of therapy.

- All "Best Practices" presentations in the AHRQ Safety Program toolkit incorporate the Four Moments framework; these slide sets can be used for presentations at conferences such as grand rounds. Most have associated One Page document templates and User Guides to assist with guideline development.

- Posters and a screen saver graphic are available that can be reproduced for posting on units and distributing to clinicians to remind them of the Four Moments. These can also be used as content for screen savers on hospital computers.

- Direct interactions by the ASP with clinicians and teams to assist them in (1) understanding the purpose of the Four Moments and (2) determining how they will be operationalized on a daily basis such as during a pre-specified time for discussion on rounds or as part of an antibiotic stewardship section of a daily progress note. The Antibiotic Time Out Tool can be used to facilitate these daily assessments by an individual or team. The ASP is encouraged to meet with frontline teams and providers to review some portion of patients receiving antibiotics; this can be guided by use of the Team Antibiotic Review Form.

Develop a Culture of Safety Around Antibiotic Prescribing

Next, as you focus on setting up or revitalizing your ASP, it is important to work on changing the culture of antibiotic prescribing at your hospital. Under the "Developing a Culture of Safety Around Antibiotic Prescribing" tab, several presentations can help you achieve this goal. Institutional behavior change can be challenging, and ASPs and frontline providers may be inclined to skip the step of addressing cultural and behavioral issues associated with antibiotic prescribing. However, we strongly recommend that the ASP team view these presentations in addition to the presentation noted above, "Making Effective

© 2021 Tamma D et al. *JAMA Network Open.*

Behavior Changes Around Antibiotic Prescribing," and determine which elements will be helpful in improving their relationship with frontline providers and in engaging frontline providers to optimize antibiotic prescribing. Below is a summary of these presentations.

- "Making the Case That Antibiotic Use is a Patient Safety Issue" provides a general overview of why improving antibiotic use is important. ASP team members should use slides from this presentation to demonstrate to leadership and frontline clinicians the potential harms associated with antibiotic use and why all individuals should work together to use antibiotics in the best possible way.
    - A signable Commitment Poster indicating to your patients and staff that your facility is dedicated to using antibiotics judiciously is available. Sign and post the Commitment Poster in public areas so that it is clear that your hospital is committed to improving antibiotic use.
- "Improving Communication and Teamwork Around Antibiotic Decision Making" addresses common pitfalls in how we communicate medical information to each other and approached to improve communication and teamwork in antibiotic prescription decisions. It includes information about initiating an antibiotic time out and use of the Team Antibiotic Review Form to accomplish these goals.
- "Identifying Targets for Improvement in Antibiotic Decision Making" addresses identifying technical versus behavioral (also known as adaptive) problems leading to antibiotic-associated adverse events as well as first and second order problem solving approaches. The ASP and frontline providers are encouraged to characterize all antibiotic prescribing problems as technical, adaptive, or both, and craft solutions based on that information in multidisciplinary teams.
- "Making Effective Changes in Antibiotic Decision Making" provides a specific framework for developing and implementing solutions to problems that lead to antibiotic associated harm. Two forms are provided to assist with these discussions between ASPs and frontline staff:
    - "Identifying Antibiotic-Associated Adverse Events Form" is brief and can be used at meetings or left on units for frontline providers to complete when they identify a potential antibiotic-associated adverse event.
    - "Learning From Antibiotic-Associated Adverse Events Form" is similar to a root cause analysis form and can be completed during structured meetings to guide strategies to prevent future antibiotic-associated adverse events.

Learn Best Practices for the Diagnosis and Treatment of Infectious Syndromes

Next, review the material under the "Best Practices for the Diagnosis and Treatment of Infectious Syndromes" tab. Each syndrome is associated with a presentation and support materials that include a One Page document that can be used as a poster, a handout, and/or as a template for local guidelines and a User Guide that assists ASPs with customizing the One Page document to reflect the local formulary and antibiogram. The specific infectious diseases topics addressed are asymptomatic

© 2021 Tamma D et al. *JAMA Network Open.*

bacteriuria and urinary tract infections, community-associated lower respiratory tract conditions (including community-acquired pneumonia, aspiration events, and chronic obstructive pulmonary disease exacerbations), ventilator-associated pneumonia, hospital-acquired pneumonia, cellulitis and skin and soft tissue abscesses, diverticulitis, biliary tract infections, bacteremia, sepsis, and *Clostridioides difficile* infections. Each presentation uses the Four Moments of Antibiotic Decision Making framework to walk the participant through relevant decisions for the specific syndrome at each moment.

The ASP should determine how to present the material to frontline providers over time. Each of the above topics includes presentation slides as well as a facilitator guide. Suggestions for presenting the material include:

- Standing monthly meetings and conferences with teams or units to review topic-specific presentations followed by the development of relevant guidelines
- Distribution of the supporting materials so that they are available at the point of care (e.g., local website, common workstations, break rooms)
- Regular follow up from the ASP with frontline staff both through routine post-prescription review and feedback and through use of the Team Antibiotic Review Form during scheduled in-person meetings. ASPs may also consider encouraging the frontline teams to review the presentations themselves

ASPs may consider focusing on a specific syndrome each month or every two months. During that period, the activities of the ASP would include developing or updating guidelines on the syndrome, disseminating information about the syndrome, focusing its daily interventions (e.g., post-prescription review and feedback and use of the Team Antibiotic Review Form) on patients with the syndrome, and collecting and feeding back data on improvements in how clinicians are managing these syndromes.

Ultimately, local guidelines for all of the covered topics as well as other topics identified by the ASP and frontline staff should be developed and made available at the point of care.

Conclusion

The AHRQ Safety Program for Improving Antibiotic Use Acute Care Toolkit provides a pathway for ASPs to develop and improve their programs. ASPs are encouraged to consider how all elements of the toolkit can be applied at their institutions to improve antibiotic use and enhance the safety of patients receiving antibiotics.

© 2021 Tamma D et al. *JAMA Network Open.*

eTable: Metrics of the Top 30 Downloaded AHRQ Safety Program Content from December 2017 to November 2018[1]

	Topic	Number of unique user downloads
1	Team antibiotic review form	923
2	Asymptomatic bacteriuria and urinary tract infection one page user guide[2]	774
3	Urinary tract infection one page document	765
4	Asymptomatic bacteriuria one page document	697
5	Antibiotic use data template	649
6	Community-acquired pneumonia one page document	648
7	Data collection and submission guide	633
8	Community-acquired pneumonia user guide	497
9	Chronic obstructive pulmonary disease exacerbation one page document	481
10	Asymptomatic bacteriuria and urinary tract infection slide set	464
11	Cellulitis one page document	432
12	*Clostridioides difficile* infection Lab-ID events data template	420
13	Aspiration pneumonitis one page document	415
14	Four moments of antibiotic decision making pocket card	395
15	Judicious Use of Antibiotics Commitment Poster	365
16	Cellulitis user guide	358
17	Antibiotic time out tool	351
18	Chronic obstructive pulmonary disease user guide	350
19	Aspiration pneumonitis user guide	344
20	Completion guide for team antibiotic review form	323
21	Community-acquired lower respiratory tract infection slide set	309
22	Four moments of antibiotic decision making posters for units	305
23	Hospital-acquired pneumonia one page document	299
24	Asymptomatic bacteriuria and urinary tract infection facilitator guide[3]	274
25	Bacteremia slide set	268
26	Ventilator-associated pneumonia webinar slide set	266
27	Diverticulitis and biliary infections webinar slide set	266
28	*Clostridiodes difficile* infections slide set	266
29	Four moments of antibiotic decision making screen savers	265
30	Sepsis slide set	260

[1]Numbers in table may be an underestimation of content utilization as many tools were also emailed to participants at the same time content was posted to the AHRQ Safety Program website.
[2]User guides describe suggested antibiotics for empiric and culture directed therapy as well as other important information to include when developing local guidelines for both children and adults.
[3]Facilitator guides include scripts to accompany the slide set used in the webinars for participants who would like to review content again or present slides locally.

© 2021 Tamma D et al. *JAMA Network Open.*

eFigure: Distribution of 402 hospitals across the United States enrolled in the AHRQ Safety Program; color gradients represent number of sites enrolled per state

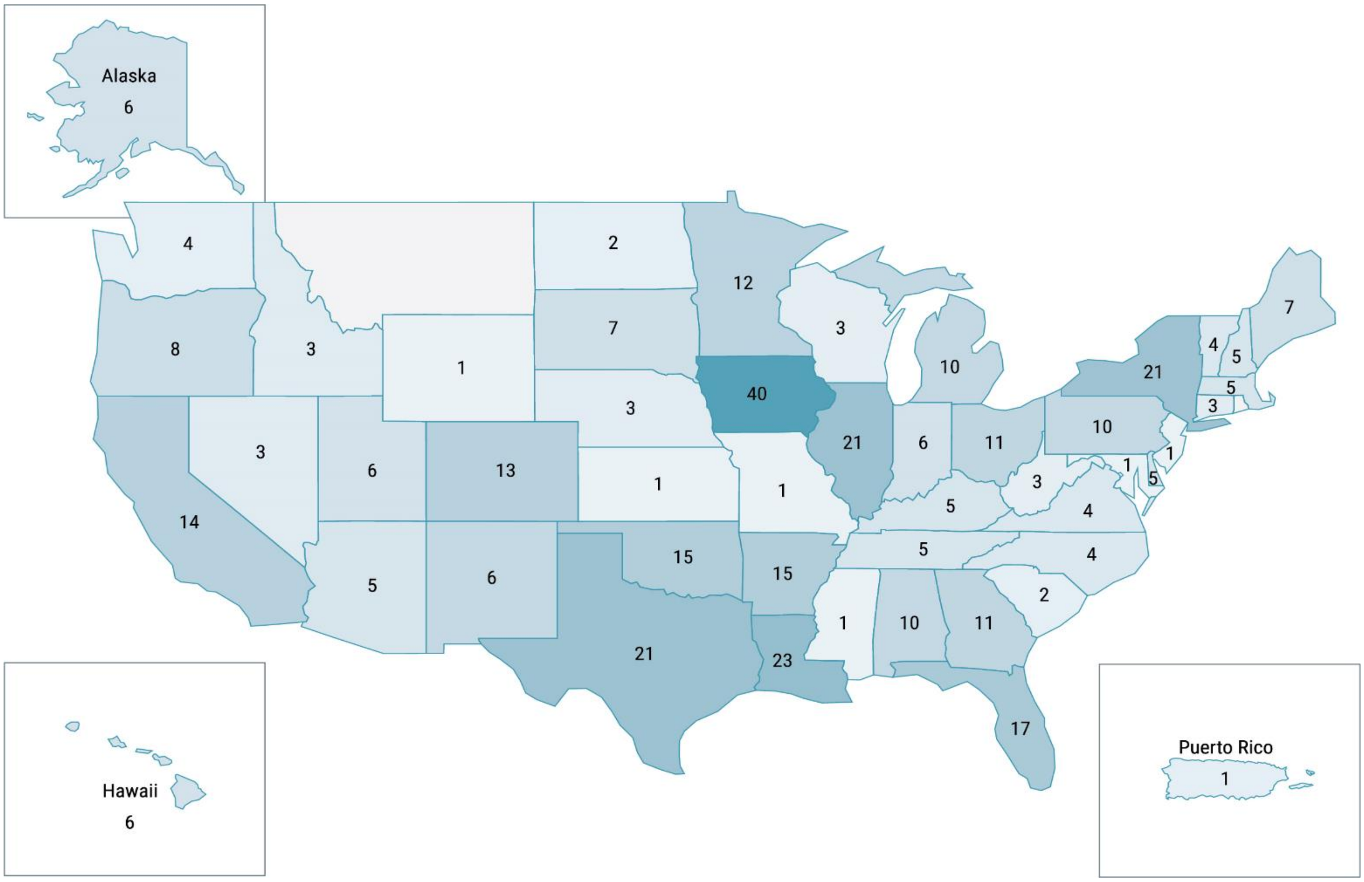

© 2021 Tamma D et al. *JAMA Network Open.*
